# Plant diversity, seasonal dynamics, and vegetation-soil relationship of Rawdhat Khuraym, Saudi Arabia: a biodiversity hotspot region within a hyper-arid region

**DOI:** 10.1186/s12870-026-09209-y

**Published:** 2026-06-08

**Authors:** Abdulaziz M. Assaeed, Asma A. Al-Huqail, Basharat A. Dar, Mariska Weijerman, Jahangir A. Malik, Abdullah S. Alharthi, Abdelhamid Elnaggar, Abdulaziz Al-Zahrani, Ahmad Alwajaan, Ahmed M. Abd-ElGawad

**Affiliations:** 1 Plant Production Department, College of Food & Agriculture Sciences, King Saud University, P.O. Box 2460, Riyadh, 11451 Saudi Arabia; 2 Chair of Climate Change, Environmental Development and Vegetation Cover, Department of Botany and Microbiology, College of Science, King Saud University, Riyadh, 11451 Saudi Arabia; 3 Imam Abudulaziz bin Mohammed Royal Reserve Development Authority, Riyadh, Saudi Arabia; 4 National Center for Vegetation Cover (NCVC), Riyadh, Saudi Arabia

**Keywords:** Vegetation hotspot zone, Rawdah ecosystem, Edaphic factor, Arid habitat, Floristic composition, *Ziziphus nummularia*

## Abstract

The Rawdah ecosystem in arid regions is characterized by its unique vegetation and biodiversity that support wildlife, buffer climate, and provide ecosystem services that benefit humans and nature. Understanding the floristic composition of the Rawdah ecosystem and its ecological dynamics is crucial for informing sustainable management. Rawdhat Khuraym, a vegetation hotspot zone in the Riyadh region of Saudi Arabia, has recently become part of the Imam Abdulaziz bin Mohammed Royal Reserve. For 23 years, the vegetation composition of this cultural and ecological important rawdhat has not been studied; hence, to inform suitable conservation and effective restoration strategies this study aims to (1) update the floristic composition of Rawdhat Khuraym in the dry and wet seasons, (2) determine the dominant plant communities within distinct parts of Rawdhat Khuraym, (3) determine the soil variables controlling the various plant communities, (4) assess the extent to which the reserve’s protection since 2020 has impacted plant communities, and (5) explore how these trajectories may alter in response to future climatic changes. Three distinct zones were identified in Rawdhat Kkuraym, and based on a random stratified sampling design, a total of 21 plots were selected. Surveys were conducted in the dry and wet season. In each plot, three replicate quadrat surveys were conducted, and soil samples were collected. For historical vegetation cover, satellite-based NDVI and climatological variables were analysed. The present study revealed a rich plant diversity in Rawdhat Khuraym, in particular, the dense growing trees and shrubs that support the plant communities in this ecosystem during the dry season, such as *Ziziphus nummularia*, *Capparis decidua*, *Vachellia. gerrardi*, *Capparis spinosa*. During the wet season, grasses like *Cynodon dactylon* and herbs like *Malva parviflora* and *Calendula arvensis* flourished after rains and became dominant. The floristic analysis showed 89 plant species (57.3% annuals and 42.4% perennials), belonging to 30 families, where Asteraceae, Poaceae, Fabaceae, and Brassicaceae were prominent (50.6%). Six life forms were recorded, with therophyte as dominant (49.4%). The Saharo-Arabian element was the most represented (55.1%), followed by the Irano-Turanian element (31.5%). In the wet winter-spring season, the northern part of Rawdhat Khuraym had three main plant communities (two dominated by *C. dactylon* and one by *Z. nummularia*). The central part also had three communities, two dominated by *M. parviflora* and one dominated by *C. arvensis*. The southern part showed four plant communities (*Zilla spinosa*, *V. gerrardi*, *Rhazya stricta*, and *C. dactylon* community). As can be expected, in the dry summer-fall season, the number of plant communities within each part became more limited; the northern part attained two communities (*Z. nummularia* and *C. spinosa*-*C. dactylon*), the central part showed one community (*C. spinosa-C. dactylon*), while the southern part had three communities (*Z. spinosa*, *V. gerrardi*, and *R. stricta*). Soil variables controlling the plant community differed in the three distinct parts. in the northern part communities showed a positive correlation with HCO_3_, Mg, Ca, salinity, SO_4_, clay, Na, NO_3_, K, porosity, field capacity, and NH_4_. In the central part, communities showed a positive correlation with silt, CaCO_3_, and pH. In the southern part, communities revealed a positive correlation with sand and the bulk density of the soil. The comparative analysis between the previous studies and the present study, 5 years after it became a protected area, revealed a recovery of perennial species, and most of the identified species are drought-resistant and show moderate salinity resistance. The NDVI analysis showed that the northern part had stable vegetation cover (average 63%) from 1986 to 2025, while central and southern parts attained lower levels of 38 and 40%, respectively. The fluctuation of NDVI can be attributed to water availability, as it showed a positive correlation with the soil wetness index, relative humidity, and precipitation. To predict future trajectories of plant communities, it is recommended to perform a detailed modelling study of the vegetation dynamics in correlation to the environmental and regional predicted future climatic conditions to be able to account for future changes in the vegetation composition of the Rawdhat Khuraym in restoration programs.

**Supplementary Information:**

The online version contains supplementary material available at 10.1186/s12870-026-09209-y.

## Introduction

Drylands cover 41% of Earth’s terrestrial surface, totaling about 5.3 million km^2^, and are home to over a third of the global population [1]. Effective management of these areas is crucial for the well-being of those people. Dryland regions are characterized by low productivity due to limited precipitation, high temperatures, low nutrient availability, and other unfavorable abiotic factors [2]. This low productivity is expected to worsen due to global climate change [3, 4], harming ecosystem functioning and, hence, reducing the ability of these ecosystems to provide essential services [5].

The sustainability of vegetation in arid environments relies mainly on plant recruitment through seeds [6]. However, constant pressure of abiotic and biotic stresses, along with low-intensity disturbances such as grazing and or other anthropogenic activities, can have pervasive effects on seedling establishment in the vegetation patches, thereby altering plant diversity and composition [7], possibly leading to the local eradication or shift of many plant species [8]. Additionally, the physicochemical attributes of the soil play an important role in influencing plant diversity. Over a specific timespan, climate change combined with anthropogenic activities may have replaced the historic community with various other communities [9, 10]. These communities could have shifted from native species to disturbance-tolerant species, followed by invasive species. In the absence of historical data, plant communities in undisturbed natural ecosystems can serve as a ‘reference ecosystem’. Natural recovery or regeneration to these reference communities depends on the level of habitat deterioration and whether the ecosystem has shifted to an alternative stable state. In some cases, assisted regeneration is necessary to enhance and facilitate recovery [11]. Such activities could be adding soil amendments to improve soil quality, restoring natural water flows, or planting missing keystone species. To better understand the appropriate reference conditions, including soil-vegetation relationships, and to identify plausible restoration and conservation targets, it is crucial to monitor changes in reference communities and conduct experimental restoration efforts on a target community, evaluating its performance in emerging climate conditions.

The vegetation cover and diversity of Saudi Arabian rangelands, though sparse, significantly contribute to the plant biodiversity of arid ecosystems worldwide [12]. These rangelands experience varying weather patterns and are primarily located in the hyper-arid climatic region, with 32.5% of the world’s hyper-arid regions located in Saudi Arabia [2]. They encompass various habitats, including rawdahs, that vary in their vegetation pattern based on the availability of moisture [13, 14]. The term “Rawdah” refers to a vegetation-rich catchment often described as a “heaven of blooming flowers”. In the spring, these regions flourish with a wide variety of mesophytic plant species [8, 15]. Rawdahs are popular for recreational activities, such as camping and herding livestock for grazing. Rawdhat Khuraym, located in the recently established Imam Abdulaziz bin Mohammed Royal Reserve (IARR), is approximately 120 km northeast of Riyadh, Saudi Arabia. One of the main objectives of this protected area is to conserve and enhance biodiversity and restore degraded habitats. Rawdhat Khuraym is an important ecological area, supporting diverse plant communities. It is also of cultural importance as it attracts numerous visitors in the winter months and was historically used for livestock grazing. Years of anthropogenic disturbances, such as off-roading, plastic pollution, logging, overgrazing, and hunting, have led to the deterioration of this Rawdah [16 and our personal field observations]. As part of national restoration initiatives, soft fences (cement blocks placed at approximately 1 m distance) were established to prevent cars from entering this Rawdah, and since the establishment of IARR in 2021, visitors are prohibited from hunting, logging, leaving rubbish, grazing livestock, or overnight camping. Understanding how the floristic composition has changed and assessing the reasons for these changes, whilst also considering the predicted impacts from climate change, will support knowledge-based management of this culturally and ecologically important area.

Based on a literature review, only a few studies about vegetation composition and floristic analysis have been recorded from Rawdhat Khuraym. A study conducted in the autumn season of 1993 by El-Din et al. [17] recorded 28 plant species forming 15 plant communities, with edaphic factors significantly influencing their distribution and abundance. Key soil parameters, such as salinity, cations, anions, and organic matter, positively influenced plant cover, while high magnesium levels were associated with decreased plant cover. In the spring season of 1995, Al-Farraj et al. [18] documented 112 species, mainly annuals (90%), with *Ziziphus nummularia*, *Vachellia tortilis*, and *Rhazya stricta* as dominant perennial species. A more detailed study on the floristic analysis of Rawdhat Khuraym was conducted from 1990 to 1999 by Alfarhan [19], where 154 plant species were identified, mainly annuals (82%). In 2003, a book on Rawdhat Khuraym was published and mentioned 115 plant species [20]. In the last 23 years, no published studies were encountered on the floristic composition and vegetation structure of Rawdhat Khuraym. A recent remote sensing study showed a significant decrease in the plant cover from 1998 to 2021, indicating its sensitivity to climate change and anthropogenic activities [21]. Based on our previous field observation of Rawdhat Khuraym, plant communities have reflected shifts from sensitive annuals to disturbance-tolerant species such as *Rhazya stricta*, and *Capparis spinosa* over the last 20 years (unpublished data).

In arid environments, understanding the factors that support restoration ecology is essential for developing sound conservation strategies, which is a significant ecological challenge, particularly concerning biological adaptation in the context of climate change [22]. To address this challenge, the conservation of the native species, biodiversity, and long-term evolutionary niches is crucial and should be a component of conservation strategies. Additionally, the cumulative stressors from climate fluctuations, anthropogenic disturbances, and resource limitations could further complicate achieving restoration objectives [23]. Therefore, designing effective restoration and conservation programs requires a thorough understanding of ecosystem attributes and processes that define these ecosystems. With the large data gap in the floristic analyses and the observed shift in species composition the main objectives of this study were to (1) assess and update the floristic composition and current vegetation structure of Rawdhat Khuraym in the dry and wet seasons, (2) determine the dominant plant communities within distinct parts of Rawdhat Khuraym, (3) assess vegetation-soil relationships and determine the soil variables controlling the various plant communities, (4) assess the extent to which the reserve’s protection since 2020 has facilitated transition toward a more stable, stress-adpated plant community, and (5) explore how these trajectories may alter in response to future climatic changes, using time-series of plant cover. The results could be used to inform management of the best restoration and conservation approaches.

## Materials and methods

### Study area

**Fig. 1:**
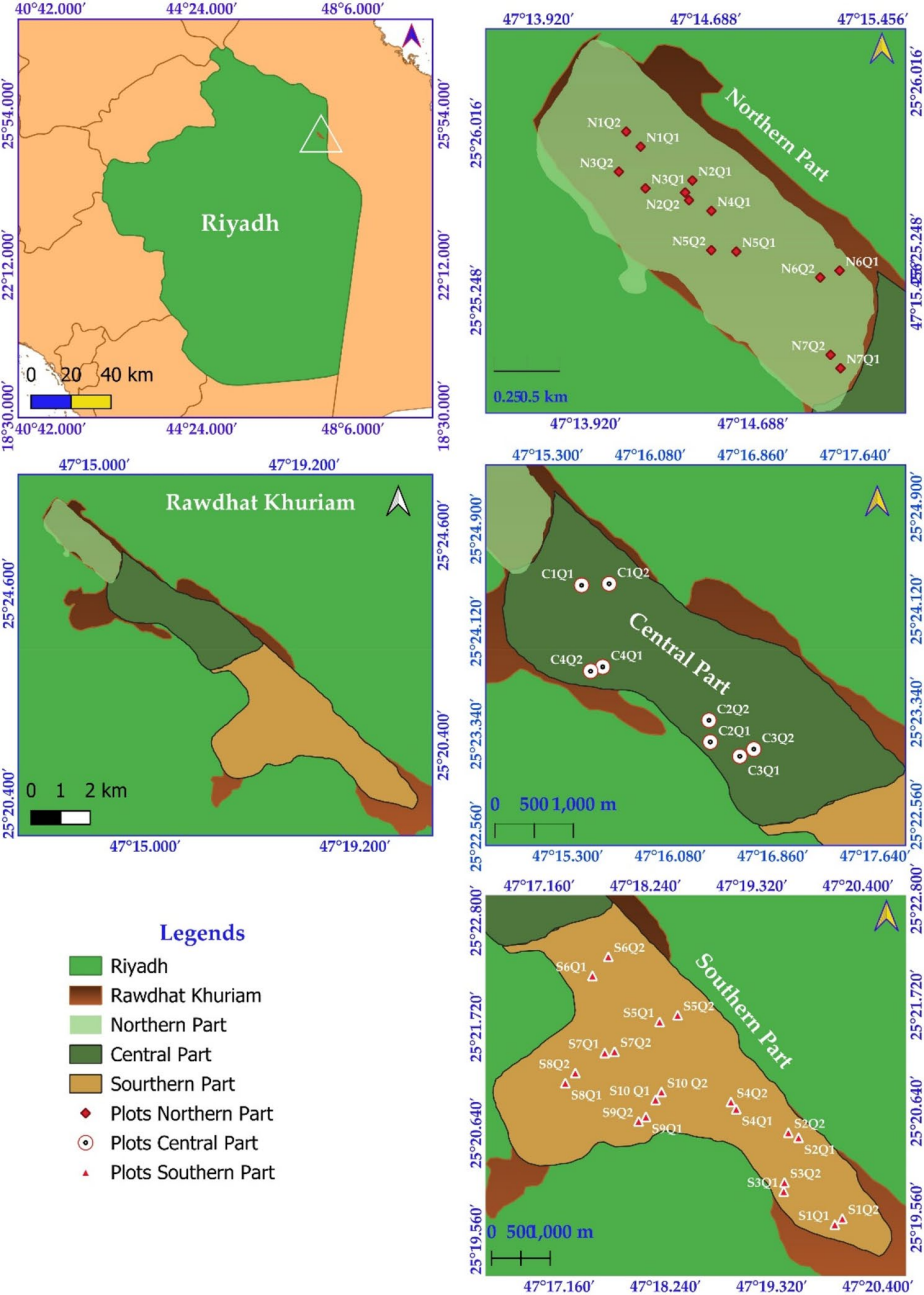
Map showing the studied area (Rawdhat Khuraym) with three ecological parts, along with the position of sampling plots and quadrats in each part

Rawdhat Khuraym, located approximately 120 km Northeast of Riyadh, Saudi Arabia, is considered the largest catchment within the protected area of Imam Abdulaziz Bin Mohammed Royal Reserve (IARR). This catchment, called “*Rawdah*” in Arabic, is approximately 19 km^2^ (Fig. 1). It is bordered by the Dahna sand belt on the east and a calcareous rocky area on the west. The surrounding topography is nearly level with a slope of less than 1%. The elevation varies from 535 to 576 m above sea level. The area consists of deep, loamy soil, which is classified as Calciorthids-Torrifluvents [24].

During the rainy season, the area, which is a natural depression, receives rainwater runoff from the upslope area to the west, channeled by various wadi (ephemeral river) systems and the deposition of fine silt. This topography creates a unique ecosystem that supports diverse and abundant flora and fauna, in contrast to the surrounding more sparsely vegetated arid environments. The ecological ecosystem of Rawdhat Khuraym is significantly influenced by its proximity to the Dahna sand belt and the Nafud Desert, the two major topographic features of the Arabian Peninsula, with their distinctive plant communities. This proximity suggests that the flora of the adjacent Dahna/Nafud ecosystems has impacted the vegetation of Rawdhat Khuraym. The soil is characterized by a range of clay to sand-rich soils. The region has an arid climate with extremely hot summers, low precipitation, and short, mild winters. The mean annual temperature exceeds 25°C. Summer temperatures can exceed 45°C, while winter temperatures range from 10°C to 20°C. The annual precipitation is 70–100 mm during the rainy season (November to April) [25]. Water retention during the rainy season makes this habitat an ecological refuge during droughts. However, during hot summers, the area experiences high evaporation rates, significantly reducing water retention. The studied area was divided into three parts (Fig. 1) based on our previous floristic surveys [26] as follows: (1) the northern part (2.78 km^2^) dominated by tall shrubs of *Ziziphus numularia* and some *Vachellia gerrardi* trees; (2) the central part with low shrubs of *Capparis spinosa* and *Calotropis procera* (2.81 km^2^); and (3) the southern part with a more mixed vegetation of acacia trees and shrubs with encroaching red sand from the adjacent Dahna desert (13.51 km^2^).

### Vegetative sampling

From December 2023 to August 2024, field trips were arranged to cover the entire area of Rawdhat Khuraym in both the dry and wet season. During the initial reconnaissance of the study area, the three distinct ecological zones: the northern part, the central part, and the southern part were demarcated (Fig. 1). This demarcation of the study area was created based on the visual differences in growth forms, vegetation patterns, topography, and soil habitat type. The survey design was based on Stratified Random Sampling to represent the ecological variability among the zones [27]. This sampling design is based on the fact that higher variability in diversity and rate of change in vegetation cover over a distance requires more intensive sampling to accurately represent the respective zones (or strata). A total of twenty-one plots were established across the three parts (Table S1). Seven random plots (N1 to N7) were established in the northern part, four plots (C1 to C4) in the central part, and 10 plots (S1 to S10) in the southern part. Sampling was conducted in all plots twice, once in the winter-spring wet season and once in the summer-fall dry season, to assess seasonal variability with respect to the species.

Within each plot, three quadrats were randomly placed with a minimum distance between them of 500 m and treated as replicates for vegetative analysis and sized according to the dominant life form. For small shrubs, grasses, and herbaceous vegetation, 10 × 10 m (100 m^2^) quadrats were used, while 30 × 30 m (900 m^2^) quadrats were used for plots with large shrubs and trees. In each quadrat, all the plant species were identified and recorded by Prof. Ahmed Abd-ElGawad (an author), a professor of plant ecology, according to the various flora of Saudi Arabia books, such as Collenette [28], Chaudhary [29], Chaudhary [30], and Chaudhary [31], and subsequently validated against the Plants of the World Online Database [32]. A plant community structure assessment within each part was conducted by calculating quantitative parameters, such as species density, percentage cover, frequency, and abundance. The plant density was determined according to Bonham [33], and the plant cover was determined using the methods of Ellenberg and Mueller-Dombois [34].

To identify the dominance for each species, the density, cover, and frequency data for each quadrat in each plot were converted into their relative values before calculating the species Importance Value Index (IVI). The average of the three quadrats was calculated. The species lifeforms were classified according to Raunkiaer [35], while the geographic distribution (chorotype) of each species was examined using various publishing materials and online data resources such as https://powo.science.kew.org and https://flora.org.il/en/plants/.

### Soil analysis

Soil samples were taken from each quadrat where vegetative sampling was conducted. Three soil samples at 0–40 cm depths were randomly collected and mixed together as one composite sample according to the standard procedure. Hence, three composite samples were collected from each plot and analysed separately as a replica. The weight-loss method was used for the determination of moisture content. The samples were duly labelled and transferred to the Range Science Laboratory, King Saud University, for further processing. In the laboratory, the samples were air-dried for a predefined time, manually crushed to break the soil aggregates into fine powder, and finally sieved through a sieve of < 2 mm pore size for subsequent analysis. Three additional soil samples were collected from each quadrat at each site to a depth of 15 cm for bulk density determination. The volume of the excavated soil was measured, and the samples were oven-dried and weighed to calculate bulk density following the method of Blake and Hartge [36]. Total soil porosity was estimated using dry bulk density values, assuming a particle density of 2.65 g/cm^3^, as described by Rowell [37], and the Bouyoucos’s method was used to determine soil texture [38]. Field capacity was measured according to Cassel and Nielsen [39]. Soil-water extract (1:5) was used for the estimation of pH and electrical conductivity (EC) according to Blake and Hartge [36]. Calcium carbonate (CaCO_3_) content was quantified using the calcimeter method [40]. The total nitrogen (N) was determined using the Kjeldahl method [41], and total phosphorus (P) spectrophotometrically by the method of Nelson and Sommers [42]. The flame photometer method (PHF 80 B biologie Spectrophotometer) was used in the determination of exchangeable sodium (Na) and potassium (K) according to Rhoades [43], while an atomic absorption spectrometer (A Perkin-Elmer, Model 2380, USA) was used to determine calcium (Ca) and magnesium (Mg) according to Allen et al. [44]. The sulphuric acid method was used to determine the Carbonate (CO_3_ and bicarbonate (HCO_3_) through titration [40]. The chloride (Cl) content was determined through the titration method using AgNO_3_ [45].

### Remote sensing data and vegetation indices

Landsat and Sentinel 2 data were used to evaluate the historical changes in vegetation cover within Rawdhat Khuraym during the period from 1986 to 2025. To account for interannual variation, five-year intervals of normalized difference vegetation index (NDVI) were used as a standard practice in remote sensing and environmental monitoring, and aligned with international reporting standards [46, 47]. The NDVI was derived from Landsat 5 data (1986 to 2000); from Landsat 7 after gap-filling the data (2001 to 2015); and from Sentinel 2 data (2016 to 2025). Cloud-free images were collected for each study period, and the median of pixel values was taken for each band to avoid outliers. NDVI was calculated under the Google Earth Engine (GEE) environment using the following equation of Rouse [48] as follows:

$$\mathrm{NDVI}=\left(\mathrm{NIR}-\mathrm{Red}\right)/\left(\mathrm{NIR}+\mathrm{Red}\right)$$

Where NIR and Red stand for near infrared and red spectral reflectance, respectively. A threshold value of 0.18 was used to distinguish between vegetated and non-vegetated pixels for each period. A binary image (two-class image) was produced for each period, and the vegetated areas and their percentages were calculated for each part of the studied area.

### Time series analysis of NDVI values

Time series of NDVI values of each part in the studied area were derived from Sentinel 2 data for December 2018 to April 2025 under the GEE environment. Climatic data of monthly precipitation, relative humidity, soil wetness, and temperature were downloaded from the NASA Prediction of Worldwide Energy Resources (POWER) data portal (https://power.larc.nasa.gov/) for the study area. If values were missing for any of the variables, the data point was removed. Pearson correlation coefficients were calculated to determine the climatic variable that had the strongest correlation with NDVI using Hmisc and broom packages in R software Ver. 4.3.2 [49]. For each part, a correlation coefficient between just rainfall and NDVI was also calculated using a 0, 1, 2, and 3 months time lag, as vegetation often has a lag time with rainfall. A time-series regression was used to analyze vegetation cover changes over the past 40 years. Statistical significance was set at *p* = 0.05.

### Data treatment

Vegetation analyses were conducted for each of the three parts of the studied area in the winter-spring and summer-fall seasons, to identify temporal and spatial variation in vegetation composition. The dataset of importance value index (IVI) of plant species in each part of the studied area was prepared to evaluate its species dominance and ecological site significance. The IVI dataset was then subjected to hierarchical cluster analysis using JMP^®^ Pro 16.0.0 (SAS Institute Inc.) to classify and determine the dominant plant communities in each part of the studied area for both seasons. To explore the ecological relationship between soil properties and vegetation composition distribution, the combined IVI dataset and soil parameter dataset were prepared and subjected to one-way ordination analysis using canonical correspondence analysis (CCA) in the MVSP software program, version 3.22 (Kovach Computing Services). This multivariate ordination method was employed to identify the fundamental gradients and environmental factors shaping the vegetation composition. To compare variation among identified plant communities and their soil traits within each part of the studied area, the raw data of the soil parameters of the plant communities of winter-spring and summer-fall were subjected to one-way ANOVA. Upon significant differences, post hoc mean comparisons were performed using Duncan’s multiple range test. The software used for one-way ANOVA was CoStat software, version 6.311, CoHort Software, 798 Lighthouse Ave. PMB 320, Monterey, CA, USA. The dataset of IVI values of all the identified species was analyzed to study the relationship among the dominant and ecologically important species and their associated soil parameters. The Pearson correlation was used to measure the correlation coefficient (r) values, which were then represented in a heatmap to demonstrate the intensity and direction of the relationship between soil properties and plant species. Furthermore, hierarchical cluster analysis was implemented to identify compositional similarity patterns and site associations. JMP^®^ Pro 16.0.0 (SAS Institute Inc., Cary, NC, USA) was used to conduct all these analyses.

## Results and discussion

### Current floristic composition of Rawdhat Khuraym

The floristic survey of the Rawdhat Khuraym revealed the presence of 89 plant species, 57.3% annuals and 42.4% perennials (Table 1). Most of the annuals finish their life cycles during short wet intervals, a strategy common in desert vegetation to avoid prolonged drought stress [50]. The recorded species belong to 30 plant families, with 50.6% of all recorded species belonging to the families Asteraceae, Poaceae, Fabaceae, and Brassicaceae (Fig. S1). None of the species recorded were listed as threatened to extinction on the IUCN Red List (Table 1). The plant species of these families are known for their drought-tolerant traits and are widely reported from arid regions in Saudi Arabia [15, 51, 52]. Based on the classification by Raunkiaer [35], six life forms were determined, where therophyte (i.e., annuals) was dominant (49.4%), followed by chamaephyte (29.2%). The prevalence of annual species is common in arid habitats [53, 54]. Moreover, these annual species have certain tactics to cope with the harsh conditions via phenotypic plasticity, genotypic diversification, and modified dispersal techniques [55, 56]. The *Orobanche pubescens* is the only parasite species among all recorded species. The chorotype analysis revealed that 61.8% of the recorded species belong to one plant geographical region, 33.7% were determined as bi-regional, and 4.5% of the species were pluri-regional. The Saharo-Arabian element was the most represented (55.1%), with 30.3% of the plant species being Saharo-Arabian mono-regional, 23.6% as bi-regional, and 1.1% as pluri-regional (Fig. S1). This distribution shows the study area’s dominating climatic and soil conditions and flora’s biogeographical compatibility with the Saharo-Arabian floristic zones [57, 58]. The second most represented element was the Irano-Turanian element (31.5%), with 20.2% being bi-regional, 6.7% mono-regional, and 4.5% pluri-regional. Among the recorded species, 22 plant species were identified as spiny plants, i.e., including spiny stems, leaves, fruits, prickles, or stipules. Only *Cakile arabica* and *Lycium shawii* were recognized as leafy succulent plants. The flowering time of each plant species is presented in Table 1.

**Table 1:** Floristic analysis of the Raudhat Khuraym site, including plant name, family, life span, life form, and chorotype, the presence of spines, plant succulence, and flowering time

Plant name	Family	Life span	Life form	Chorotype	Spinescence	Succulence	Flowering time	IUCN category
*Acacia salicina* Lindl.	Fabaceae	Perennial	Ph	Aus	No	No	Jun-Oct	LC
*Achillea fragrantissima* (Forssk.) Sch.Bip.	Asteraceae	Perennial	He	SA, IT	No	No	Mar-Sep	NE
*Aerva javanica* (Burm.f.) Juss. ex Schult.	Amaranthaceae	Perennial	Ch	Tr	No	No	Dec-Jun	NE
*Anisosciadium lanatum* Boiss.	Apiaceae	Annual	Ch	IT, SA	No	No	Mar-May	NE
*Anthemis deserti* Boiss.	Asteraceae	Annual	Th	SA, M	No	No	Mar-May	NE
*Asphodelus tenuifolius* Cav.	Liliaceae	Annual	Th	SA, Su	No	No	Feb-Apr	NE
*Astragalus crenatus* Schult.	Fabaceae	Annual	Th	SA, IT	No	No	Mar-Apr	NE
*Astragalus spinosus* (Forssk.) Muschl.	Fabaceae	Perennial	Ch	IT	Leaves	No	Feb-Apr	NE
*Atractylis cancellata* L.	Asteraceae	Annual	Th	M	Leaves	No	Feb-May	NE
*Avena fatua* L.	Poaceae	Annual	Th	Tr	No	No	Mar-Apr	LC
*Blepharis attenuata NapP*	Acanthaceae	Perennial	Ch	IT, SA	Leaves	No	Apr-Jun	NE
*Bromus tectorum* L.	Poaceae	Annual	Th	M, IT	No	No	Mar-Apr	NE
*Cakile arabica* Velen. & Bornm.	Brassicaceae	Annual	Th	SA	No	Leaf	Dec-Feb	NE
*Calendula arvensis* L.	Asteraceae	Annual	Th	M, IT	No	No	Dec-Apr	NE
*Calotropis procera* (Aiton) Aiton f.	Asclepiadaceae	Perennial	Ch	Su	No	No	Mar-Nov	LC
*Capparis decidua* (Forssk.) Edgew.	Capparaceae	Perennial	Ph	Su	Stipules	No	Apr-Sep	LC
*Capparis spinosa* L.	Capparaceae	Perennial	Ch	M	Stipule	No	Apr-Sep	LC
*Carthamus oxyacanthus* M.Bieb.	Asteraceae	Annual	Ch	SA	Leaf	No	Apr-Aug	NE
*Cenchrus ciliaris* L.	Poaceae	Perennial	He	SA, Su	No	No	Jan-Dec	LC
*Centaurea sinaica* DC.	Asteraceae	Annual	Th	SA	Bracts	No	Apr-May	NE
*Chrozophora tinctoria* (L.) Raf.	Euphorbiaceae	Annual	Th	Tr	No	No	Apr-Oct	LC
*Citrullus colocynthis* (L.) Schrad.	Cucurbitaceae	Annual	Th	SA	No	No	Feb-May	NE
*Cleome amblyocarpa* Barratte & Murb.	Capparaceae	Annual	Th	SA, Su	No	No	Mar-July	NE
*Coincya tournefortii* (Gouan) Alcaraz, T.E.Díaz	Brassicaceae	Annual	Th	SA, M	No	No	Dec-Apr	NE
*Convolvulus cephalopodus* Boiss.	Convolvulaceae	Perennial	Ph	SA	No	No	Mar-Apr	NE
*Convolvulus oxyphyllus* Boiss.	Convolvulaceae	Perennial	Ph	SA	No	No	Mar-Apr	NE
*Convolvulus pilosellifolius* Desr.	Convolvulaceae	Annual	Th	IT	No	No	Feb-Jun	NE
*Cynodon dactylon (L.) Ps.*	Poaceae	Perennial	Ge	Cosm	No	No	Apr-Dec	NE
*Echium rauwolfii* Delile	Boraginaceae	Annual	Th	SA	Leaf, Stem	No	Mar-Apr	NE
*Eleusine indica* (L.) Gaertn.	Poaceae	Annual	Th	Tr	No	No	Jun-Nov	LC
*Ephedra ciliata* Fisch. & C.A.Mey.	Ephedraceae	Perennial	Ch	SA	No	No	Mar-Jun	LC
*Erigeron bonariensis* L.	Asteraceae	Annual	Th	Am	No	No	Apr-Oct	NE
*Erodium laciniatum* (Cav.) Willd.	Geraniaceae	Annual	Th	SA	No	No	Feb-Apr	NE
*Euphorbia granulata* Forssk.	Euphorbiaceae	Annual	Th	Su	No	No	Feb-Mar	NE
*Fagonia indica* Burm.f.	Zygophyllaceae	Perennial	Ch	SA	Stipules	No	Mar-May	NE
*Farsetia aegyptia* Turra	Brassicaceae	Perennial	Ch	Su	No	No	Jan-May	NE
*Filago desertorum* Pomel	Asteraceae	Annual	Th	SA, IT	No	No	Feb-May	NE
*Forsskaolea tenacissima* L.	Urticaceae	Perennial	Ch	SA, Su	Prickles	No	Jan-Apr	NE
*Gypsophila capillaris* (Forssk.) C.Chr.	Caryophyllaceae	Perennial	Ch	IT	No	No	Apr-Nov	NE
*Haplophyllum tuberculatum* (Forssk.) A.Juss.	Rutaceae	Perennial	Ch	SA	No	No	Jan-Dec	NE
*Heliotropium ramosissimum* (Lehm.) Sieber ex DC.	Boraginaceae	Perennial	Ch	SA	No	No	Mar-Apr	NE
*Hordeum murinum* L.	Poaceae	Annual	Th	SA, M	No	No	Mar-May	LC
*Lactuca serriola* L.	Asteraceae	Annual	Th	ES, M, IT	No	No	Jun-Oct	NE
*Lasiurus scindicus* Henrard	Poaceae	Perennial	He	Su	No	No	Jan-Nov	NE
*Launaea angustifolia* (Desf.) Kuntze	Asteraceae	Annual	Th	SA	No	No	Feb-Apr	NE
*Launaea capitata* (Spreng.) Dandy	Asteraceae	Annual	Th	SA	No	No	Mar-Apr	NE
*Launaea nudicaulis* (L.) Hook.f.	Asteraceae	Perennial	He	SA	No	No	Jan-May	NE
*Launaea procumbens* (Roxb.) Ramayya & Rajagopal	Asteraceae	Annual	Ch	SA	No	No	Mar-Apr	NE
*Lolium rigidum* Gaudin	Poaceae	Annual	Ch	M, IT	No	No	Feb-May	NE
*Lycium shawii* Roem. & Schult.	Solanaceae	Perennial	Ph	SA, Su	Stem	Leaf	Nov-Jun	LC
*Malva neglecta Wallr.*	Malvaceae	Annual	Th	ES, M, IT	No	No	Feb-Jun	NE
*Malva parviflora* L.	Malvaceae	Annual	Th	M, IT	No	No	Feb-May	NE
*Medicago laciniata* (L.) Mill.	Fabaceae	Annual	Th	SA	No	No	Feb-May	NE
*Moltkiopsis ciliata* (Forssk.) I.M.Johnst.	Boraginaceae	Perennial	Ch	SA	No	No	Feb-May	NE
*Neurada procumbens* L.	Neuradaceae	Annual	Th	SA	Fruits	No	Feb-Apri	NE
*Notoceras bicorne* (Aiton) Amo	Brassicaceae	Annual	Th	SA	No	No	Dec-Mar	NE
*Orobanche pubescens* d’Urv.	Orobanchaceae	Annual	P	SA, M, IT	No	No	Mar-Jun	NE
*Otoglyphis factorovskyi* (Warb. & Eig) Oberpr. & Vogt	Asteraceae	Annual	Th	Su	No	No	Mar-Jun	NE
*Paronychia arabica (L.) DC.*	Caryophyllaceae	Annual	He	IT	No	No	Feb-May	NE
*Phalaris minor* Retz.	Poaceae	Annual	Th	M, IT	No	No	Feb-Jun	NE
*Picris cyanocarpa* Boiss.	Asteraceae	Annual	Th	SA	No	No	Mar-Apr	NE
*Plantago albicans* L.	Plantaginaceae	Perennial	He	SA, M	No	No	Mar-May	NE
*Plantago amplexicaulis* Cav.	Plantaginaceae	Annual	Th	SA	No	No	Mar-Apr	NE
*Plantago ovata* Forssk.	Plantaginaceae	Annual	Th	SA, IT	No	No	Jan-Apr	NE
*Polycarpaea repens* (Forssk.) Asch. & Schweinf.	Caryophyllaceae	Perennial	He	Su	No	No	Mar-Apr	NE
*Polygonum aviculare* L.	Polygonaceae	Annual	Th	M, IT	No	No	Mar-Jul	NE
*Prosopis farcta* (Banks & Sol.) J.F.Macbr.	Fabaceae	Perennial	Ch	IT	Prickles	No	Mar-Aug	LC
*Pulicaria undulata* (Forssk.) C.A.Mey.	Asteraceae	Perennial	Ch	SA, Su	No	No	Jan-Mar	NE
*Reseda aucheri* Boiss.	Resedaceae	Annual	Th	M, IT	No	No	Feb-Apr	NE
*Rhanterium epapposum* Oliv.	Asteraceae	Perennial	Ch	SA, IT	No	No	Nov-Jan	NE
*Rhazya stricta* Decne.	Apocynaceae	Perennial	Ch	SA, Su	No	No	Dec-Mar	NE
*Rumex spinosus* L.	Polygonaceae	Annual	Th	M	Fruit	No	Nov-Apr	NE
*Salvia aegyptiaca* L.	Lamiaceae	Perennial	Ch	SA	No	No	Jan-Apr	NE
*Salvia spinosa* L.	Lamiaceae	Annual	He	IT	No	No	Mar-Jun	NE
*Schismus arabicus* Nees	Poaceae	Annual	Th	SA	No	No	Mar-Apr	NE
*Schismus barbatus* (L.) Thell.	Poaceae	Annual	Th	SA, IT	No	No	Mar-Apr	NE
*Sisymbrium irio* L.	Brassicaceae	Annual	Th	M, IT	No	No	Jan-May	NE
*Stipellula capensis* (Thunb.) Röser & Hamasha	Poaceae	Annual	Th	SA, IT	No	No	Mar-May	NE
*Teucrium oliverianum* Ging. ex Benth.	Lamiaceae	Perennial	Ch	Cosm	No	No	Mar-May	NE
*Teucrium polium* L.	Lamiaceae	Perennial	Ch	M, IT	No	No	Apr-Aug	NE
*Tribulus terrestris L.*	Zygophyllaceae	Annual	Th	ES, M, IT	Fruit	no	Mar-Oct	LC
*Trigonella stellata* Forssk.	Fabaceae	Annual	Th	SA	No	No	Feb-Apr	NE
*Vachellia farnesiana* (L.) Wight & Arn.	Fabaceae	Perennial	Ph	Tr	Stem	No	Feb-Mar	LC
*Vachellia flava* (Forssk.) Kyal. & Boatwr.	Fabaceae	Perennial	Ph	SA-Su	Stipule	No	Feb-Apr	LC
*Vachellia gerrardi* (Benth.) P.J.H.Hurter	Fabaceae	Perennial	Ph	SZ	Stipule	No	Jun-Dec	NE
*Xanthium spinosum* L.	Asteraceae	Annual	Th	Tr	Fruits	No	Jun-Sep	NE
*Zilla spinosa* (L.) Prantl	Brassicaceae	Perennial	Ch	SA	Stem	No	Jan-May	NE
*Ziziphus nummularia* (Burm.f.) Whigt & Arnott	Rhamnaceae	Perennial	Ph	Su	Stipule	No	Jun-Jul	LC
*Zygophyllum bruguieri* (DC.) Christenh. & Byng	Zygophyllaceae	Perennial	Ch	SA	Stipule	No	Feb-Apr	NE

*Th* therophytes, *Ch* chamaephytes, *Ph* phanerophytes, *He *hemicryptophytes, *G* geophytes, and *P* parasites. *Am* American, *ES* Euro-Siberian, *Tr* Tropical, *Cosm* Cosmopolitan, *Aus* Australian, *Su* Sudanian, *M* Mediterranean, *IT* Irano-Turanian, *SA * Saharo-Arabian, *Am* American, *SZ* Sudano-Zambezian, *LC* least concern, *NE* not evaluated

No identified species are determined as endemic species. Only 16 species (17.98%) of all identified species have been assessed and determined as “Least Concerned” according to the IUCN Red List of threatened species (Table 1). These species are *Acacia salicina*, *Avena fatua*, *Calotropis procera*, *Capparis decidua*, *Capparis spinosa*, *Cenchrus ciliaris*, *Chrozophora tinctoria*, *Eleusine indica*, *Ephedra ciliata*, *Hordeum murinum*, *Lycium shawii*, *Prosopis farcta*, *Tribulus terrestris*, *Vachellia farnesiana*, *Vachellia flava*, and *Ziziphus nummularia*.

### Comparative analysis with previous studies on plant diversity in Rawdhat Khuraym

**Fig. 2:**
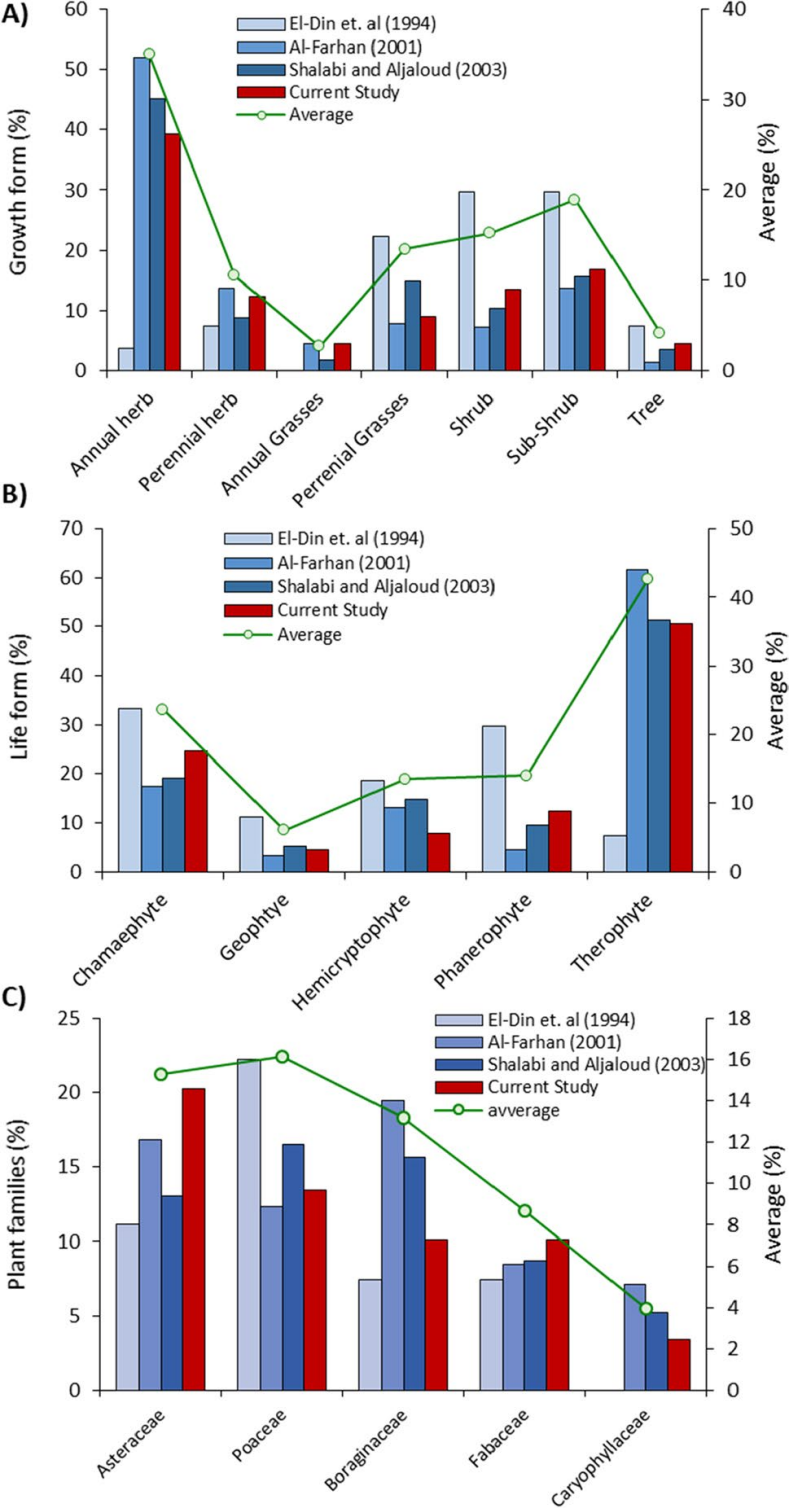
Comparative analysis of the growth forms (**A**) and life forms (**B**), and plant families (**C**) of the plant species recorded in Rawdhat Khuraym based on the previous studies and the current study

Based on the literature review, we found three previously published works on the plant diversity of Rawdhat Khuraym. El-Din et al. [17] described the daphic factors influencing the plant communities of Rawdhat Khuraym in 1993. Alfarhan [19] assessed the floristic composition from 1990 to 1999. In 2003, Shalbi and Aljoloud [20] describe the ecology and phytosociology of Rawdhat Khuraym. No information on the plant composition or spatial distribution was provided in these publications. A floristic information dataset of 213 plant species was compiled from the three identified publications and the results of the present study, and includes plant species names, families, growth forms, life forms, drought-resistant, salt-resistant, and ecological significance (Table S2). The annual herbaceous (herb) growth form is the dominant form within Rawdhat Khuraym according to all studies, represented by 35.0% of all species (Fig. 2A), while the lowest represented growth form is the annual grasses (2.7%). Overall, in 1993, perennial species were more dominant compared to the annual species, while the subsequent studies (1999 and 2003) showed less perennial species, particularly the shrubs and trees. The current study reveals the recovery of perennial species, for example, the tree life form is recovered by 71.1 and 16.6% compared to studies of Alfarhan [19] and Shalbi and Aljoloud [20], respectively. This trend could be ascribed to the rainfall factor, where the average rainfall showed the same trend. The annual species in desert ecosystems flourish quickly after rainfall and complete their life cycle within a few months to escape from dry periods [52, 59, 60]. The same trend for life forms was observed in the analyzed data from previous studies and the current study (Fig. 2B). The Poaceae, Asteraceae, Boraginaceae, and Fabaceae were the most represented families within Rawdhat Khuraym in all four studies, with slight differences in the dominant families among the studies (Fig. 2C).

**Fig. 3:**
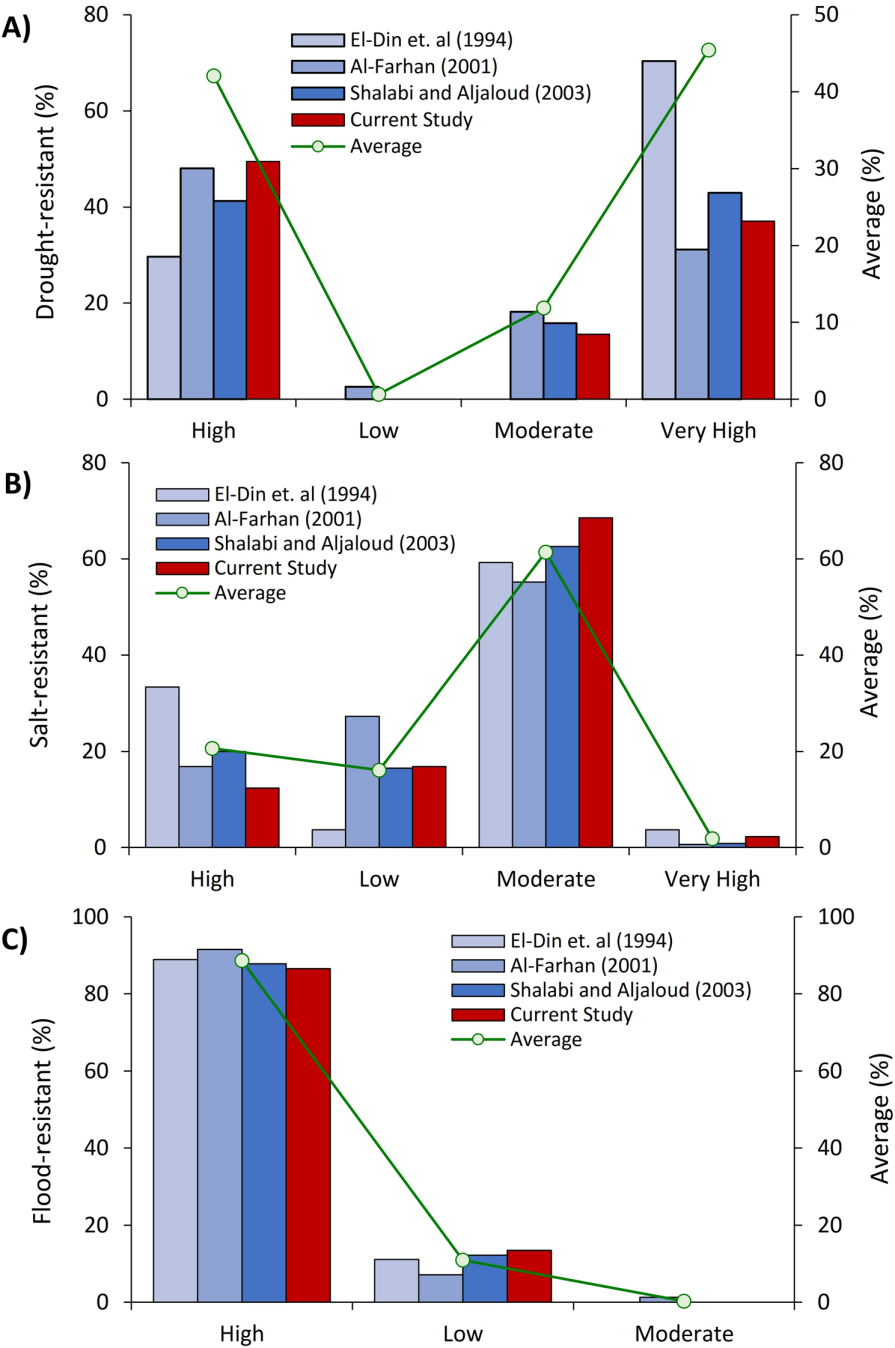
Comparative analysis of the drought-resistant (**A**), salt-resistant (**B**), and flood-resistant (**C**) plant species recorded in Rawdhat Khuraym based on the previous studies and the current study

As can be expected in an arid ecosystem, 87.5% of all recorded plants from the four studies are identified as “high” or “very-high” drought-resistant species, while only 0.6% of the plant species have low resistance to drought (Fig. 3A). Additionally, no considerable variation was observed between the previous studies and the current study. On the other hand, most of the recorded plant species (61.4%) have been identified as moderately salt-resistant species (Fig. 3B). The soil of the studied area is not saline (0.2–1.2 dS/m), as it is a catchment area of the water coming via wadis (ephemeral river valleys) that funnel water to the catchment area. The majority of the identified plants of the Rawdhat Khuraym can also be classified as flood-tolerant species (88.7%), indicating their adaptation to the topography and the nature of the study area as “Rawdhat” (Fig. 3C). After rainfall, water collects in the Rawdhat ecosystem, resulting in temporary ponds that support lush vegetation and recharge the groundwater [8, 54].

### Current plant communities in the winter-spring season

The vegetation of the Rawdhat Khuraym in the winter-spring season showed distinct plant communities within each part (Table 2), based on the hierarchical cluster analysis of the IVI (Fig. S2). In the northern part, three plant communities were present. The first community was dominated by the grass *Cynodon dactylon* (IVI = 86.6), followed by *Ziziphus nummularia*, *Vachellia gerrardi*, and *Polycarpaea repens*, with IVI values of IVI of 41.8, 18.3, and 15.9, respectively. Similar to the first community, the second community of the northern part was also dominated by *Cynodon dactylon* (IVI = 107.7), but associated with *Malva parviflora* (IVI = 33.8), *Calotropis procera* (IVI = 25.4), and *Capparis spinosa* (IVI = 22.7). The third community was dominated by the shrubs of *Ziziphus nummularia* (IVI = 53.1), and associated with the species *Vachellia gerrardi*, *Polycarpaea repens*, *Carthamus oxyacanthus*, and *Capparis decidua*, with IVIs of 29.8, 28.4, 33.8, and 11.7, respectively. The dominance of *Cynodon dactylon* across two communities with different associated species shows its competitive advantage and adaptability to different environmental and disturbance conditions.

**Table 2:** Plant communities and diversity indexes of Rawdhat Khuraym, Saudi Arabia, during the winter-spring and summer-fall seasons

Season	Part	Community	Species No.	Dominant species	Important species
Winter-Spring	Northern	Com1 (N1, N2, N4)	16	*Cynodon dactylon* [86.6]*	*Ziziphus nummularia* [41.8], *Vachellia gerrardi* [18.3], *Polycarpaea repens* [15.9]
		Com2 (N6, N7)	12	*Cynodon dactylon* [107.7]	*Malva parviflora* [33.8], *Calotropis procera* [25.4], *Capparis spinosa* [22.7]
		Com3 (N3, N5)	13	*Ziziphus nummularia* [53.1]	*Vachellia gerrardi* [29.8], *Polycarpaea repens* [28.4], *Carthamus oxyacanthus* [33.8], *Capparis decidua* [11.7]
	Central	Com1 (C1)	16	*Malva parviflora* [56.1]	*Capparis spinosa* [47.1], *Cynodon dactylon* [47.0]
		Com2 (C2, C3)	22	*Malva parviflora* [90.1]	*Rhazya stricta* [26.7], *Rumex spinosus* [23.4], *Cynodon dactylon* [12.9]
		Com3 (C4)	25	*Calendula arvensis* [62.3]	*Plantago ovata* [44.0], *Malva parviflora* [28.9], *Capparis spinosa* [19.0]
	Southern	Com1 (S1, S3)	19	*Zilla spinosa* [61.2]	*Achillea fragrantissima* [22.7], *Salvia aegyptiaca* [19.3], *Pulicaria undulata* [18.6]
		Com2 (S4)	12	*Vachellia gerrardi* [77.6]	*Convolvulus oxyphyllus* [68.3], *Lycium shawii* [22.7], *Ziziphus nummularia* [17.4]
		Com3 (S2, S5, C9, S10)	39	*Rhazya stricta* [46.1]	*Phalaris minor* [32.5], *Heliotropium ramosissimum* [30.8], *Vachellia gerrardi* [22.0]
		Com4 (C6, C7, C8)	18	*Cynodon dactylon* [83.5]	*Vachellia gerrardi* [37.1], *Lycium shawii* [22.5]
Summer-Fall	Northern	Com1 (N1, N2, N3, N4, N5)	15	*Ziziphus nummularia* [54.8]	*Cynodon dactylon* [45.0], *Vachellia gerrardi* [29.9], *Capparis decidua* [16.1]
		Com2 (N6, N7)	17	*Capparis spinosa* [53.8] *Cynodon dactylon* [53.0]	*Calotropis procera* [38.9], *Phalaris minor* [14.4], *Avena fatua* [11.5]
	Central	Com1 (C1)	3	*Cynodon dactylon* [179.3]	*Carthamus oxyacanthus* [18.9]
		Com2 (C2, C3, C4)	31	*Cynodon dactylon* [35.2] *Capparis spinosa* [35.0]	*Rhazya stricta* [26.4], *Carthamus oxyacanthus* [19.8], *Pulicaria undulata* [19.8]
	Southern	Com1 (S1, S3)	14	*Zilla spinosa* [75.4]	*Pulicaria undulata* [22.4], *Stipellula capensis* [20.1], *Convolvulus pilosellifolius* [16.6], *Lycium shawii* [16.1], *Rhazya stricta* [16.1]
		Com2 (S2, S4, S6, S7, S8)	21	*Vachellia gerrardi* [162.9]	*Lycium shawii* [65.8], *Cynodon dactylon* [31.2], *Ephedra ciliata* [19.8]
		Com3 (S5, C9, S10)	17	*Rhazya stricta* [115.5]	*Lycium shawii* [31.9], *Vachellia gerrardi* [12.4]

* importance value index

The central part of the Rawdhat Khuraym also showed three plant communities, two of them dominated by the annual herb *Malva parviflora* (IVI = 56.1 and 90.1), but one with an association of *Capparis spinosa* (IVI = 47.1) and *Cynodon dactylon* (IVI = 47.0), while the other community was associated with *Rhazya stricta* (IVI = 26.7), *Rumex spinosus* (IVI = 23.4), and *Cynodon dactylon* (IVI = 12.9). The third community was dominated by the herb *Calendula arvensis* (IVI = 62.3), with an association of *Plantago ovata*, *Malva parviflora*, and *Capparis spinosa*, with IVIs of 44.0, 28.9, and 19.0, respectively. *Malva parviflora* and *Calendula arvensis* species are fast-growing annual species that exploit the short wet season to complete their life cycles, an adaptive strategy widely observed in desert annual flora [61].

The vegetation in the southern part of the Rawdhat Khuraym had the highest structural complexity and harboured four plant communities. The first community was dominated by the sub-shrub *Zilla spinosa*, with an IVI of 61.2. In this community, other important plant species were *Achillea fragrantissima*, *Salvia aegyptiaca*, and *Pulicaria undulata*, with an IVI of 22.7, 19.3, and 18.6, respectively (Table 2). The second community was dominated by the trees of *Vachellia gerrardi*, with an association of the herb *Convolvulus oxyphyllus and* the shrubs of *Lycium shawii* and *Ziziphus nummularia*. These woody shrub species are essential for the long-term stability of arid ecosystems by facilitating nutrient cycling and supporting drought tolerance through their deep roots, which allow water infiltration and increase the water content under the canopy [62]. The third community consisted of *Rhazya stricta* associated with *Phalaris minor*, *Helioropium ramosissimum*, and *Vachellia gerrardi*. The fourth community was again dominated by the grass *Cynodon dactylon*, which attained an IVI of 83.5 and had associated tree species of *Vachellia gerrardi* and *Capparis decidua*.

### Current plant communities in the summer-fall season

The plant communities in the summer-autumn seasons were more limited due to the lower presence of annual plants (Table 2). Within the northern part, two plant communities were identified. The first community was dominated by the shrubs of *Ziziphus nummularia*, and associated with the important species *Cynodon dactylon*, *Vachellia gerrardi*, and *Capparis decidua*. The second community was co-dominated by *Capparis spinosa* and *Cynodon dactylon*, and the important species *Calotropis procera* and the grasses *Phalaris minor* and *Avena fatua*. The shift toward a shrub-dominated community co-occurring with disturbance-tolerant species such as *Capparis spinosa* confirms a seasonal successional shift in vegetation pattern.

The central part of Rawdhat Khuraym also showed two plant communities. The first community was strongly dominated by *Cynodon dactylon* ( IVI = 179). The second plant community was co-dominated by *Cynodon dactylon* and *Capparis spinosa*, with an association of the important plants *Rhazya stricta*, *Carthamus oxyacanthus*, and *Pulicaria undulata*. The southern part of Rawdhat Khuraym showed similar plant communities compared with the winter-spring season; the dominant species were the same in the three communities, indicating that the perennial plant species were more dominant in the southern part throughout the year. The first community was still dominated by the sub-shrubs of *Zilla spinosa*, with an association of *Pulicaria undulata*, *Stipellula capensis*, *Convolvulus pilosellifolius*, *Lycium shawii*, and *Rhazya stricta*. The second community, dominated by the trees of *Vachellia gerrardi*, which attained a high IVI of 163. This community is associated with *Lycium shawii*, *Cynodon dactylon*, and *Ephedra ciliata*. The last community dominated by the *Rhazya stricta*, had an association of *Lycium shawii* and *Vachellia gerrardi* (Table 2). Disturbance-tolerant *Rhazya stricta* might survive extreme environmental stress by using its allolopahtic property of specialized chemical defense and physiological adaptations [63].

### Vegetation-soil relationship of the winter-spring season

The soil analysis of the samples of each plant community is shown in Table 3. The soils of the plant communities in the northern part of Rawdhat Khuraym showed comparable characteristics, except for the soil texture, pH, NO_3_, and NH_4_, which showed a significant variation (*P* < 0.05). However, the communities of the central part showed significant variation for most of the analyzed soil properties, except for bulk density, porosity, Mg, HCO_3_, and NO_3_. Finally, the southern part of Rawdhat Khuraym showed significant variation among the identified communities regarding the soil texture, field capacity, bulk density, porosity, pH, salinity, Na, Ca, NH_4_, and available phosphorus (Table 3).

**Table 3:** The soil variables of the identified plant communities in Rawdhat Khuraym during the winter-spring season

Variable	Northern part			*P*-value	Central part			*P*-value	Southern part				*P*-value
	Com1	Com2	Com3		Com1	Com2	Com3		Com1	Com2	Com3	Com4	
Sand (%)	24.58 ± 1.19^a^	13.75 ± 2.12^b^	16.88 ± 5.09^b^	0.0219*	22.50 ± 5.00^ab^	34.38 ± 12.05^a^	12.50 ± 0.00^b^	0.0298*	45.00 ± 3.06^a^	30.00 ± 7.50^b^	56.56 ± 4.20^a^	27.50 ± 4.43^b^	< 0.001***
Silt (%)	36.50 ± 2.25^c^	58.13 ± 2.26^a^	49.38 ± 4.81^b^	0.002***	46.25 ± 3.75^b^	43.13 ± 9.86^b^	61.25 ± 3.75^a^	0.175*	31.25 ± 2.98^ab^	35.00 ± 2.50^ab^	23.75 ± 3.81^b^	42.08 ± 5.14^a^	0.0279*
Clay (%)	38.92 ± 2.02^a^	28.13 ± 1.28^c^	33.75 ± 1.02^b^	0.002***	31.25 ± 1.25^a^	22.50 ± 2.70^c^	26.25 ± 3.75^b^	0.003***	23.75 ± 1.61^c^	35.00 ± 5.00^b^	19.69 ± 1.67^c^	30.42 ± 2.18^b^	< 0.001***
FC (%)	31.95 ± 0.36^a^	31.99 ± 0.40^a^	32.51 ± 0.82^a^	0.6814^ns^	31.03 ± 1.22^a^	27.98 ± 2.11^b^	31.51 ± 0.01^a^	0.0351*	26.66 ± 0.52^b^	30.56 ± 1.69^a^	24.30 ± 0.86^b^	29.86 ± 0.58^a^	< 0.001***
BD (g/cm^3^)	1.44 ± 0.00^a^	1.44 ± 0.00^a^	1.44 ± 0.01^a^	0.9572^ns^	1.45 ± 0.01^a^	1.46 ± 0.02^a^	1.45 ± 0.00^a^	0.2304^ns^	1.48 ± 0.00^a^	1.46 ± 0.01^b^	1.49 ± 0.010^a^	1.46 ± 0.01^b^	< 0.001***
Porosity (%)	44.18 ± 0.17^a^	43.90 ± 0.12^a^	43.87 ± 0.18^a^	0.3299^ns^	43.86 ± 0.27^a^	43.13 ± 0.63^a^	43.87 ± 0.05^a^	0.1480^ns^	42.39 ± 0.25^b^	43.53 ± 0.42^a^	41.96 ± 0.20^b^	43.63 ± 0.18^a^	< 0.001***
pH	6.96 ± 0.05^b^	7.34 ± 0.08^a^	7.10 ± 0.11^b^	0.0021***	7.57 ± 0.03^a^	7.55 ± 0.13^ab^	7.36 ± 0.08^b^	0.0357*	7.40 ± 0.04^a^	7.28 ± 0.17^b^	7.49 ± 0.04^a^	7.24 ± 0.03^b^	0.0001***
EC (dS/m)	0.86 ± 0.14^a^	0.56 ± 0.09^a^	0.89 ± 0.26^a^	0.2487^ns^	0.31 ± 0.03^a^	0.21 ± 0.02^c^	0.27 ± 0.02^b^	< 0.001***	0.24 ± 0.02^b^	0.32 ± 0.02^b^	0.21 ± 0.01^b^	0.49 ± 0.08^a^	0.0005***
Na (meq/L)	0.61 ± 0.08^a^	0.42 ± 0.05^a^	0.66 ± 0.12^a^	0.1111^ns^	0.47 ± 0.09^a^	0.30 ± 0.04^b^	0.18 ± 0.08^c^	< 0.001***	0.12 ± 0.06^bc^	0.42 ± 0.06^a^	0.05 ± 0.02^c^	0.26 ± 0.01^ab^	0.0016**
K (meq/L)	1.30 ± 0.22^a^	1.19 ± 0.26^a^	1.12 ± 0.38^a^	0.7508^ns^	0.47 ± 0.02^b^	0.33 ± 0.04^c^	0.57 ± 0.02^a^	< 0.001***	0.33 ± 002^b^	0.39 ± 0.06^ab^	0.31 ± 0.04^b^	0.73 ± 0.23^a^	0.0767^ns^
Ca (meq/L)	6.93 ± 0.98^a^	3.85 ± 0.44^a^	7.45 ± 2.48^a^	0.1369^ns^	2.10 ± 0.10^a^	1.20 ± 0.47^b^	2.00 ± 0.00^b^	0.0024**	2.10 ± 0.13^b^	2.30 ± 0.10^b^	1.75 ± 0.12^b^	3.90 ± 0.36^a^	< 0.001***
Mg (meq/L)	2.63 ± 0.37^a^	1.80 ± 0.49^a^	2.20 ± 0.55^a^	0.3259^ns^	0.80 ± 0.00^a^	0.95 ± 0.45^a^	1.00 ± 0.20^a^	0.5964^ns^	1.20 ± 0.60^a^	1.50 ± 0.30^a^	1.45 ± 0.61^a^	1.00 ± 0.14^a^	0.6957^ns^
SO_4_^2^ (meq/L)	7.07 ± 0.70^a^	3.60 ± 0.71^a^	6.95 ± 2.48^a^	0.1138^ns^	2.00 ± 0.20^a^	1.65 ± 0.69^b^	3.10 ± 0.50^a^	0.0114*	2.40 ± 0.08^a^	2.80 ± 0.04^a^	1.88 ± 0.15^a^	2.43 ± 0.59^a^	0.2926^ns^
HCO_3_ (meq/L)	1.52 ± 0.64^a^	1.25 ± 0.27^a^	2.48 ± 0.74^a^	0.1836^ns^	0.55 ± 0.25^b^	0.58 ± 0.06^ab^	0.70 ± 0.02^a^	0.0787^ns^	0.80 ± 0.12^a^	1.25 ± 0.05^a^	1.03 ± 0.27^a^	1.52 ± 0.43^a^	0.3907^ns^
Cl (meq/L)	5.02 ± 2.17^a^	2.35 ± 0.56^a^	1.35 ± 0.29^a^	0.1476^ns^	1.90 ± 1.10^a^	2.45 ± 0.49^a^	0.50 ± 0.30^b^	0.002***	2.35 ± 0.57^a^	1.30 ± 0.30^a^	2.08 ± 0.27^a^	2.30 ± 0.76^a^	0.3709^ns^
CaCO_3_ (%)	33.96 ± 1.61^a^	41.94 ± 1.04^a^	32.78 ± 4.81^a^	0.0860^ns^	36.16 ± 1.77^b^	36.08 ± 4.93^b^	43.87 ± 1.45^a^	0.0236*	23.22 ± 1.48^a^	26.19 ± 3.37^a^	23.18 ± 4.12^a^	33.16 ± 2.93^a^	0.0771^ns^
K (mg/kg)	50.92 ± 8.42^a^	46.53 ± 10.32^a^	43.65 ± 14.76^a^	0.7527^ns^	18.45 ± 0.95^b^	12.95 ± 1.71^c^	22.35 ± 0.95^a^	< 0.001***	12.93 ± 0.93^b^	15.20 ± 2.30^ab^	12.10 ± 1.73^b^	28.35 ± 8.92^a^	0.0736^ns^
NO_3_ (mg/kg)	2.26 ± 0.50^b^	3.18 ± 0.49^ab^	4.76 ± 0.63^a^	0.0106*	1.41 ± 0.43^b^	1.90 ± 0.94^ab^	2.71 ± 0.39^a^	0.0692^ns^	1.78 ± 0.68^a^	1.80 ± 0.35^a^	2.04 ± 0.18^a^	2.21 ± 0.36^a^	0.7507^ns^
NH_4_^+^ (mg/kg)	41.11 ± 7.90^b^	74.46 ± 13.47^a^	36.81 ± 8.61^b^	0.0104*	41.92 ± 8.93^a^	22.63 ± 3.81^b^	27.45 ± 20.85^b^	0.0144*	14.83 ± 4.88^b^	4.06 ± 3.69^c^	11.75 ± 2.36^b^	34.07 ± 3.98^a^	< 0.001***
Av. P (mg/kg)	31.89 ± 3.88	31.59 ± 1.83	33.19 ± 2.82	0.9514^ns^	28.38 ± 8.51^a^	18.35 ± 3.26^b^	19.59 ± 0.04^b^	0.0026**	14.31 ± 0.72^b^	20.86 ± 2.36^b^	15.27 ± 1.20^b^	26.82 ± 3.98^a^	0.007***

The different superscript letters among plant communities of each season showed a value significant at a probability level of 0.05.

*BD* Bulk density,* FC* Field capacity, *Av. P * Available phosphorus

# values are average ± standard error

* *p* < 0.05, ** *p* < 0.01, ns: *p* > 0.05

**Fig. 4:**
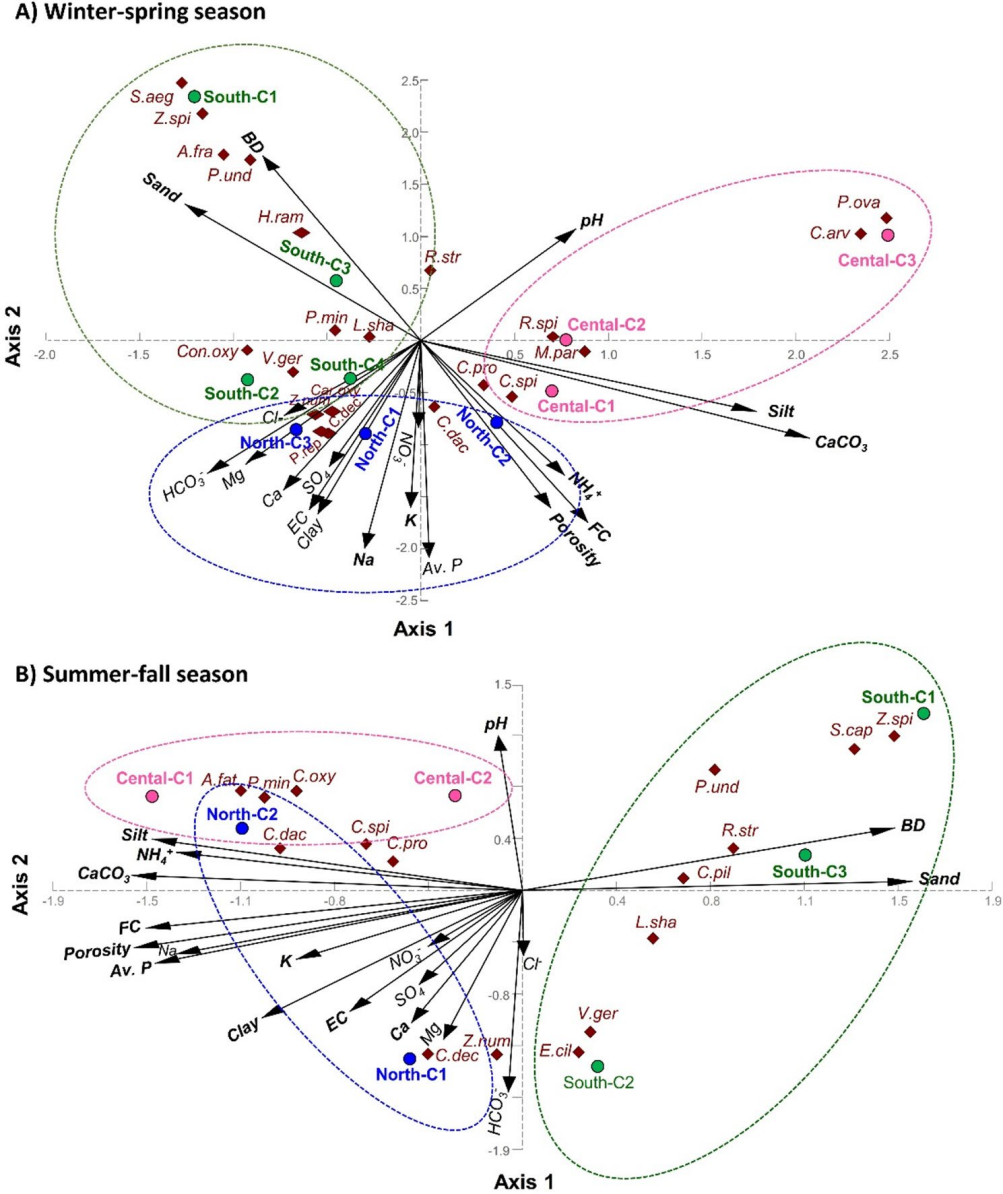
The Canonical Correlation Analysis (CCA) of the dominant and important plant species of each community and the soil properties of the winter-spring season (**A**) and summer-fall season (**B**). A.fra: *Achillea fragrantissima*, A.fat: *Avena fatua*, C.arv: *Calendula arvensis*, C.pro: *Calotropis procera*, C.dec: *Capparis decidua*, C.spi: *Capparis spinosa*, Car.oxy: *Carthamus oxyacanthus*, Con.oxy: *Convolvulus oxyphyllus*, C.pil: *Convolvulus pilosellifolius*, C.dac: *Cynodon dactylon*, E.cil: *Ephedra ciliata*, H.ram: *Heliotropium ramosissimum*, L.sha: *Lycium shawii*, M.par: *Malva parviflora*, P.min: *Phalaris minor*, P.ova: *Plantago ovata*, P.rep: *Polycarpaea repens*, P.und: *Pulicaria undulata*, R.str: *Rhazya stricta*, R.spi: *Rumex spinosus*, S.aeg: *Salvia aegyptiaca*, S.cap: *Stipellula capensis*, V.ger: *Vachellia gerrardi*, Z.spi: *Zilla spinosa*, and Z.num: *Ziziphus nummularia*

The application of the Canonical Correspondence Analysis (CCA) on the dataset of the importance values of the dominant and important species, as well as the soil characteristics, showed the correlation among the plant communities and soil properties (Fig. 4). The communities of each part in Rawdhat Khuraym clustered together with minimal overlap among them, confirming the presence of the distinct parts of Rawdhat Khuraym. The northern part communities showed moderate correlations with most of the soil properties (HCO_3_, Mg, Ca, salinity, SO_4_, clay, Na, NO_3_, K, porosity, field capacity, and NH_4_), while the species in central part communities showed both weak (e.g., *Calotropis procera* and *Capparis spinosa*) and strong (*Calendula arvensis* and *Plantago ovata*) positive correlations to the silt content, CaCO_3_, and pH. The species in the southern part communities revealed weak (e.g., *Lycium shawii*) to moderate (e.g., *Heliotropium ramosissimum*) to strong (e.g., *Zilla spinosa* and *Salvia aegyptica*) correlations to the sand content and bulk density of the soil.

**Fig. 5:**
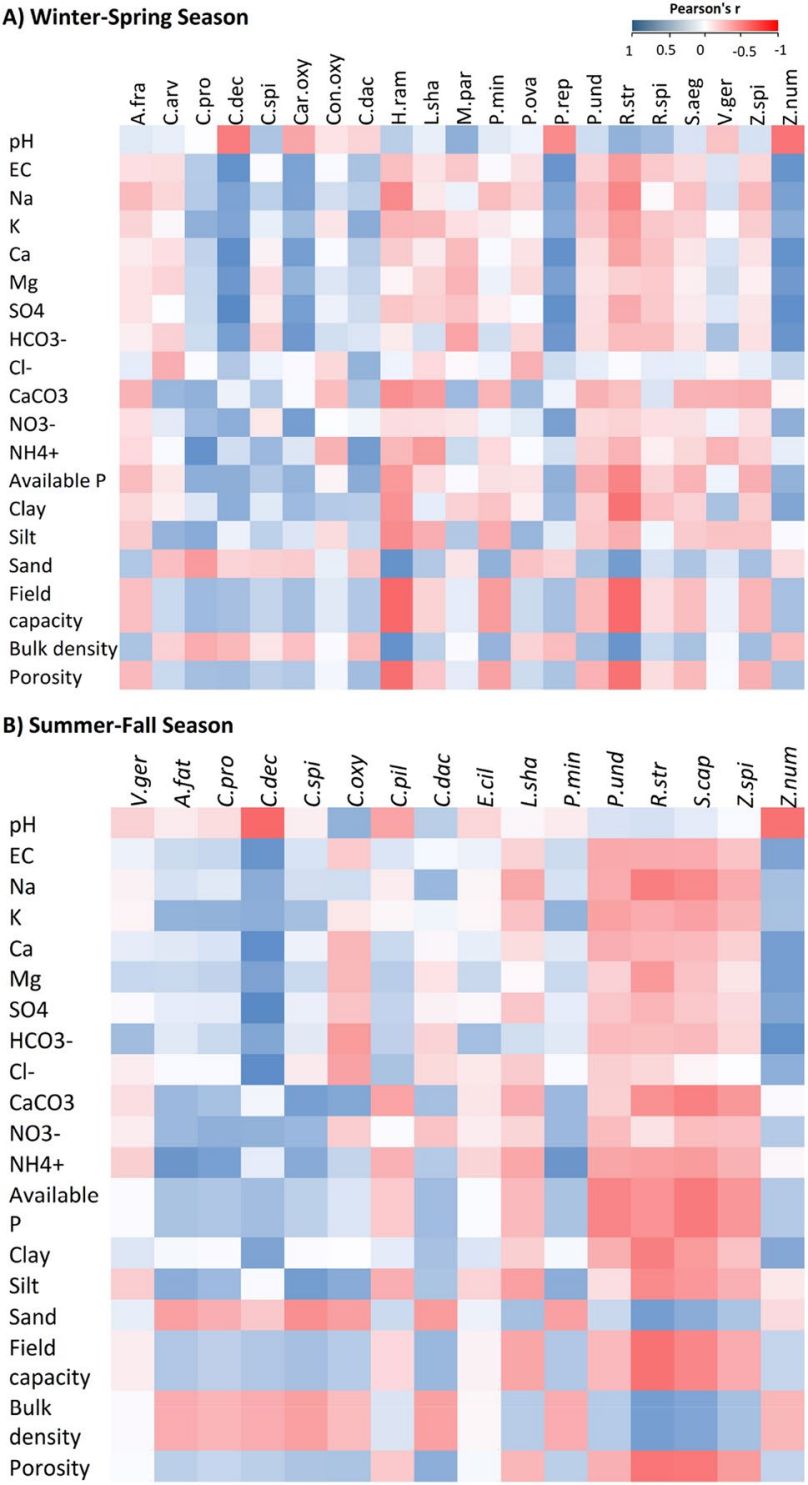
The Pearson correlation coefficient (r) heatmap of the dominant and important plant species of each community and the various soil variables of the winter-spring season (A) and summer-fall season (B). A.fra: *Achillea fragrantissima*, A.fat: *Avena fatua*, C.arv: *Calendula arvensis*, C.pro: *Calotropis procera*, C.dec: *Capparis decidua*, C.spi: *Capparis spinosa*, Car.oxy: *Carthamus oxyacanthus*, Con.oxy: *Convolvulus oxyphyllus*, C.pil: *Convolvulus pilosellifolius*, C.dac: *Cynodon dactylon*, E.cil: *Ephedra ciliata*, H.ram: *Heliotropium ramosissimum*, L.sha: *Lycium shawii*, M.par: *Malva parviflora*, P.min: *Phalaris minor*, P.ova: *Plantago ovata*, P.rep: *Polycarpaea repens*, P.und: *Pulicaria undulata*, R.str: *Rhazya stricta*, R.spi: *Rumex spinosus*, S.aeg: *Salvia aegyptiaca*, S.cap: *Stipellula capensis*, V.ger: *Vachellia gerrardi*, Z.spi: *Zilla spinosa*, and Z.num: *Ziziphus nummularia*

Regarding species that were dominant in various communities and parts, such as, *Cynodon dactylon* (dominant species of communities 1 & 2 of the northern part and the dominant species of community 4 of the southern part), they showed a strong positive correlation to K, NH_4_, and available phosphorus (Fig. 5 and Table S3), while they revealed a strong negative correlation to the bulk density (*r* = -0.44). Like all species in the northern part, the shrubs of *Ziziphus nummularia*, which were the dominant species of community 3 of the northern part, showed a positive correlation with most of the soil parameters (Na, K, Ca, Mg, SO_4_, HCO_3_, NO_3_, clay content, and salinity), while they showed a strong negative correlation to the pH (*r* = -0.83) (Figs. 4 and 5 and Table S3). *Z. nummularia* have been reported as a salinity- and drought-tolerant plant [64] because of their adaptive mechanisms toward drought, via the up-regulation of genes responsible for maintaining water potential, abscisic acid biosynthesis, sugar metabolism, osmoregulation, and reactive oxygen species control [64, 65]. In this context, *Z. nummularia* has been reported as a nurse/facilitator plant for other plants via the regulation of the microhabitat and nutrient enrichment in the soil [66].

The *Rhazya stricta* (dominant species of community 3 in the southern part), *Heliotropium ramosissimum*, and *Phalaris minor* (important species of community 3 in the southern part), as well as the *Zilla spinosa* (dominant species of community 1 in the southern part), *Achillea fragrantissima*, *Salvia aegyptiaca* (important species of community 1 in the southern part), and *Pulicaria undulata* (important species of community 1 in the southern part), showed a strong to moderate positive correlation (*r* > 0.40) with the bulk density and sand content, while they showed a strong negative correlation to the available phorphorus, Na, CaCO_3_, filed capacity, and porosity (Figs. 4 and 5). This pattern indicates that these species preferred the low salinity and sandy habitats [15, 52].

The trees of *Vachellia gerrardi* (the dominant species of communities 2 of the southern part, the important species of community 1 and 3 of the northern part, and the important species of communities 3 and 4 of the southern part) showed a strong positive correlation to the HCO_3_ (*r* = 0.46) and clay content (*r* = 0.45), while it showed a strong negative correlation to the CaCO_3_ (*r* = -0.49) and NH_4_ (*r* = -0.48).

### Vegetation-soil relationship of the summer-fall season

The various soil parameters of each identified plant community during the summer-fall season are presented in Table 4. The two identified plant communities in the northern part of Rawdhat Khuraym showed significant variation (*P* < 0.05) for silt, clay, pH, Ca, SO_4_, and NH_4_; however, the two identified plant communities in the central part showed significant variation in Na, NH_4_, available phosphorus, clay, and salinity. The southern part plant communities revealed significant variation (*P* < 0.05) for most of the analyzed soil variables (sand, clay, field capacity, bulk density, porosity, salinity, Na, Ca, HCO_3_, and available phosphorus).

**Table 4:** The soil variables of the identified plant communities in Rawdhat Khuraym during the summer-fall season

Variable	Northern part		*P*-value	Central part		*P*-value	Southern part			*P*-value
	Com1	Com2		Com1	Com2		Com1	Com2	Com3	
Sand (%)	21.50^a^±2.69	13.75^a^±2.60	0.1120ns	22.50 ± 5.00^a^	27.08 ± 8.91^a^	05593^ns^	45.00 ± 3.06^ab^	34.75 ± 5.62^b^	55.00 ± 4.28^a^	0.0123*
Silt (%)	41.65 ± 3.28^b^	58.13 ± 2.77^a^	0.0137*	46.25 ± 3.75^a^	49.17 ± 7.38^a^	0.7726^ns^	31.25 ± 2.98^ab^	36.25 ± 4.29^a^	25.00 ± 4.70^b^	0.1134
Clay (%)	36.85 ± 1.51^a^	28.13 ± 1.57^b^	0.0023**	31.25 ± 1.25^a^	23.75 ± 2.12^b^	0.0048**	23.75 ± 1.61^ab^	29.00 ± 2.51^a^	20.00 ± 1.58^b^	0.0173*
FC (%)	32.17 ± 0.43^a^	31.99 ± 0.49^a^	0.4900^ns^	31.03 ± 1.22^a^	29.16 ± 1.53^a^	0.2087^ns^	26.66 ± 0.52^ab^	28.68 ± 1.13^a^	24.66 ± 0.72^b^	0.0091**
BD (g/cm^3^)	1.44 ± 0.00^a^	1.44 ± 0.01^a^	0.7208^ns^	1.45 ± 0.01^a^	1.46 ± 0.01^a^	0.5199^ns^	1.48 ± 0.00^ab^	1.46 ± 0.01^b^	1.49 ± 0.00^a^	0.0094**
Porosity (%)	44.06 ± 0.14^a^	43.90 ± 0.15^a^	0.3497^ns^	43.86 ± 0.27^a^	43.38 ± 0.43^a^	0.2515^ns^	42.39 ± 0.25^b^	43.24 ± 0.30^a^	42.01 ± 0.17^b^	0.0030**
pH	7.01 ± 0.06^b^	7.34 ± 0.09^a^	0.0170*	7.57 ± 0.03^a^	7.48 ± 0.09^a^	0.3442^ns^	7.40 ± 0.04	7.32 ± 0.06	7.45 ± 0.04	0.1124
EC (dS/m)	0.87 ± 0.14^a^	0.56 ± 0.11^a^	0.0995^ns^	0.31 ± 0.03^a^	0.23 ± 0.02^b^	0.0012**	0.24 ± 0.02^b^	0.39 ± 0.06^a^	0.22 ± 0.01^b^	0.0337*
Na (meq/L)	0.63 ± 0.07^a^	0.42 ± 0.07^b^	0.0261*	0.47 ± 0.09^a^	0.26 ± 0.04^b^	0.0012**	0.12 ± 0.06^ab^	0.26 ± 0.07^a^	0.03 ± 0.01^b^	0.0431*
K (meq/L)	1.23 ± 0.21^a^	1.19 ± 0.32^a^	0.8910^ns^	0.47 ± 0.02^a^	0.41 ± 0.06^a^	0.3062^ns^	0.33 ± 0.02	0.55 ± 0.15	0.36 ± 0.04	0.2546
Ca (meq/L)	7.14 ± 1.25^a^	3.85 ± 0.54^b^	0.0295*	2.10 ± 0.10^a^	1.47 ± 0.34^a^	0.0854^ns^	2.10 ± 0.13^b^	3.06 ± 0.41^a^	1.90 ± 0.10^b^	0.0214*
Mg (meq/L)	2.46 ± 0.33^a^	1.80 ± 0.61^a^	0.4643^ns^	0.80 ± 0.00^a^	0.97 ± 0.29^a^	0.5775^ns^	1.20 ± 0.60	1.68 ± 0.45	0.63 ± 0.14	0.2420
SO_4_^2^ (meq/L)	7.02 ± 1.18^a^	3.60 ± 0.87^b^	0.0485*	2.00 ± 0.20^a^	2.13 ± 0.55^a^	0.8671^ns^	2.40 ± 0.08^ab^	2.42 ± 0.35^a^	1.83 ± 0.19^b^	0.0585
HCO_3_ (meq/L)	1.90 ± 0.52^a^	1.25 ± 0.33^a^	0.3519^ns^	0.55 ± 0.25^a^	0.62 ± 0.07^a^	0.2804^ns^	0.80 ± 0.12^b^	1.55 ± 0.29^a^	0.72 ± 0.08^b^	0.0007***
Cl (meq/L)	3.55 ± 1.40^a^	2.35 ± 0.68^a^	0.6628^ns^	1.90 ± 1.10^a^	1.80 ± 0.52^a^	0.6215^ns^	2.35 ± 0.57	1.98 ± 0.47	2.20 ± 0.34	0.9792
CaCO_3_ (%)	33.49 ± 2.35^a^	41.94 ± 1.28^a^	0.0622^ns^	36.16 ± 1.77^a^	38.67 ± 3.55^a^	0.5550^ns^	23.22 ± 1.48^b^	30.44 ± 2.98^a^	22.07 ± 4.13^b^	0.0554
K (mg/kg)	48.01 ± 8.28^a^	46.53 ± 12.64^a^	0.8885^ns^	18.45 ± 0.95^a^	16.08 ± 2.27^a^	0.2911^ns^	12.93 ± 0.93	21.34 ± 5.98	13.98 ± 1.58	0.2525
NO_3_ (mg/kg)	3.26 ± 0.57^a^	3.18 ± 0.60^a^	0.8170^ns^	1.41 ± 0.43^a^	2.17 ± 0.63^a^	0.2165^ns^	1.78 ± 0.68	2.01 ± 0.23	2.18 ± 0.19	0.6161
NH_4_^+^ (mg/kg)	39.39 ± 6.01^b^	74.46 ± 16.49^a^	0.0205*	41.92 ± 8.93^a^	24.24 ± 5.98^b^	0.0328*	14.83 ± 4.88	22.38 ± 5.93	13.79 ± 2.23	0.1434
Av. P (mg/kg)	32.41 ± 2.58^a^	31.59 ± 2.25^a^	0.8647^ns^	28.38 ± 8.51^a^	18.76 ± 2.08^b^	0.0279*	14.31 ± 0.72^b^	22.82 ± 2.94^a^	16.10 ± 1.37^b^	0.0232*

The different superscript letters among plant communities of each season showed a value significant at a probability level of 0.05

*BD* Bulk density, *FC* Field capacity, *Av. P* Available phosphorus

# values are average ± standard error

* *p* < 0.05, ** *p* < 0.01, ns: *p* > 0.05

The application of the CCA on the combined datasets of the soil variables and the dataset of importance values of the dominant and important species showed overlap of the second community of the northern part with communities in the central part (Fig. 4B), while the communities in the southern part clustered separately. The shrubs of *Ziziphus nummularia* (the dominant species of community 1 of the northern part), as well as the trees of *Capparis decidua* (important species of community 1 of the northern part), showed a strong positive correlation with most of the soil parameters (Ca, Mg, Na, K, SO_4_, Cl, HCO_3_, salinity, NH_4_, and Clay), while both species showed a strong negative correlation to the pH (Fig. 5 and Table S4). The observed overlap between the northern and central part of Rawdhat Khoraym is driven by the subshrubs of *Capparis spinosa* (the dominant species of community 2 of the northern part and the dominant species of community 2 in the central part), as well as the trees of *Calotropis procera* (an important species of community 2 of the northern part), showed a strong (*r* > 0.60) to moderate (0.40 < *r* > 0.60) significant correlation to K, CaCO_3_, NO_3_, NH_4_, available phosphorus, and silt content, while they showed a significant negative correlation to the sand fraction and the bulk density of the soil (Tables S3). The grass *Cynodon dactylon*, which is the dominant species in community 3 of the northern part, community 1 and 2 in the central part, revealed a similar positive correlation with NH_4_, available phosphorus, Na, CaCO_3_, but, in this season, also with clay, silt, field capacity, and porosity of the soil. Similar to the winter-spring results, it showed a negative correlation with bulk density (*r* = -0.53) of the soil and, in this season, also with sand content (*r* = -0.56). *Rhazya stricta* (a dominant species of community 3 and an important species in community 1 of the southern part, and an important species in community 2 in the central part), showed a strong positive correlation with sand fraction (*r* = 0.80) and bulk density (*r* = 0.79) of the soil (Fig. 5 and Table S4), and a moderate to strong negative correlation to most of soil parameters (field capacity, porosity, salinity, Na, K, Ca, Mg, SO_4_, CaCO_3_, NH_4_, available phorsphorus, clay, and silt) similar to the winter-spring season.

### Vegetation cover changes and time series

**Fig. 6:**
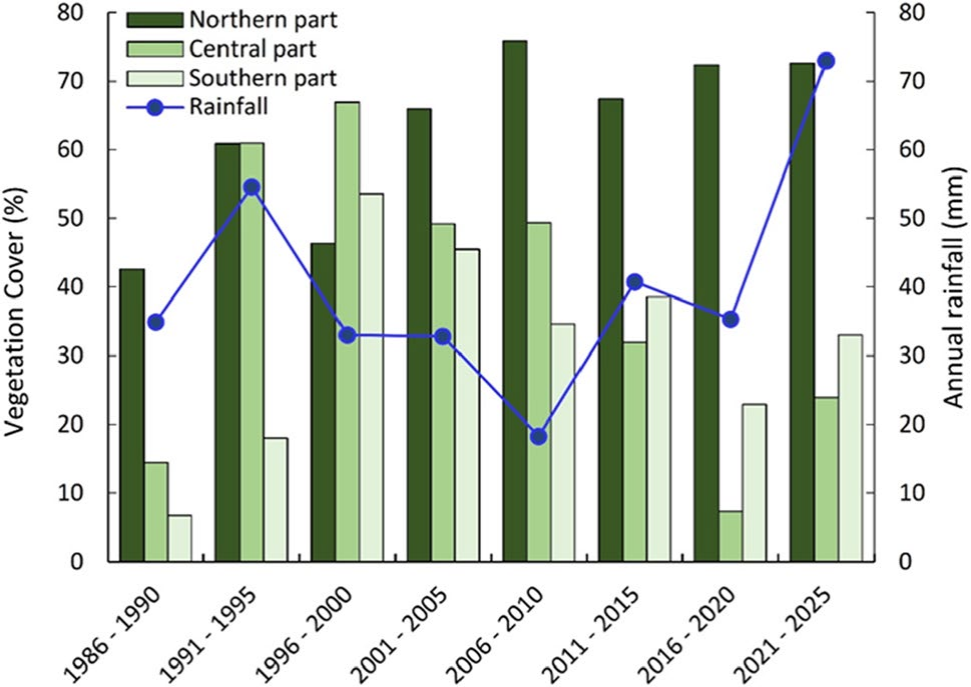
Changes in vegetation cover and rainfall within the studied area from 1986 to 2025

The three parts of Rawdhat Khuraym showed a different pattern in vegetation cover from 1986 to 2025 (Fig. 6). The northern part had a relatively high vegetation cover of 40% in the late 1980s, which increased and stabilized at around 80% from the early 2000s onwards. The central and southern parts had a low vegetation cover in the late 1980s, but increased in cover in the early (central part) and late (southern part) 1990s. However, since the 2000s, vegetation cover has generally decreased in these two areas, possibly related to the decrease in rainfall. From the 2006–2010 period onwards, despite an increase in rainfall, vegetation did not recover and decreased further until the early 2020s (Fig. 6). Only in these last 5 years, recovery is apparent, which coincides with the peak in rainfall as well as with the establishment of the protected area of Imam Abdulaziz bin Mohamed Royal Reserve, which prohibits grazing. The spatial distribution of vegetation cover in these 5-year time blocks is illustrated in Figs. S3-S6.

**Fig. 7:**
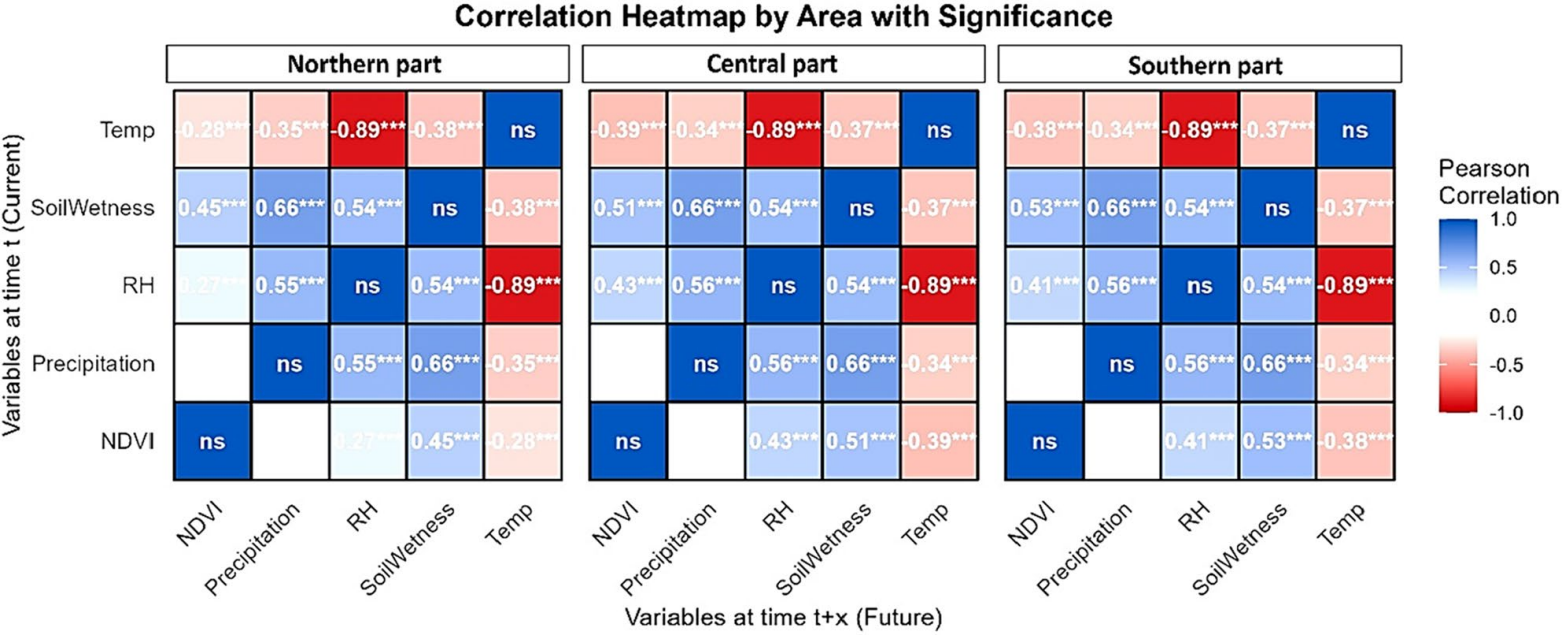
Correlation heatmap for the three areas among NDVI and climatic variables. *** *p* < 0.001, ns = non-significant, RH: relative humidity

Time series analysis of NDVI values and monthly precipitation in the studied areas from December 1986 to April 2025 showed highly significant correlations between the winter rain and NDVI after a one to two-month time lag (Table 5). Consequently, a strong response in vegetation cover is evident after winter rains (Fig. S7). This could be due to the emergence of predominantly annual plants and, secondly, to the perennials growing and blossoming [59], consistent with the results of the vegetation analyses. It was evident from analyses of the four climatic variables (temperature, soil wetness index, relative humidity, and precipitation (with lag-times) that soil wetness index had the highest significant and positive correlation with NDVI (Fig. 7). Temperature had negative correlations with all other variables, and the strongest negative correlation was with relative humidity. On the other hand, the soil wetness index showed the strongest positive correlation with precipitation, followed by relative humidity and NDVI.

**Table 5:** Correlation between NDVI values derived from landsat data from 1986 to 2025 and rainfall and at 0, 1, 2- and 3-months lag times. The strongest correlations are shown in bold for each area

Lag time	Northern part	Central part	Southern part
No lag time	(r= 0.14, p< 0.001)	(r= 0.17, p< 0.001)	(r= 0.20, p< 0.001)
One month	(r= 0.30, p< 0.001)	( **r=0.40**,p< 0.001)	(r= 0.37, p< 0.001)
Two months	(**r= 0.38**, p< 0.001)	(r= 0.39, p< 0.001)	(**r= 0.38**, p< 0.001)
Three months	(r= 0.33, p< 0.001)	(r= 0.20, p< 0.001)	(r= 0.24, p< 0.001)

The temperature in the Arabian Peninsula, the Middle East, and the Northern Africa region has increased up to three times faster than the global average [67]. Under the worst-case emission scenario (SSP5-8.5; CO_2_ emissions triple by 2075), the temperature in the Arabian Peninsula is predicted to increase by 7 °C. Rainfall is also expected to increase, with the central part of Saudi Arabia likely receiving 10–40% more rain [68]. To predict future vegetation cover, we can use the identified strong correlation between NDVI and soil wetness. To this end, we calculated the linear regression coefficients using the equation: NDVI = Intercept + Slope × Soil Wetness. Based on climate model predictions of soil wetness, the expected change in NDVI can theoretically now be predicted to inform management of Rawdhat Khuraym. However, predicted rain episodes are expected to be less frequent with much higher intensities. This phenomenon could result in intense runoff combined with soil erosion and low water infiltration, and hence less water available to stimulate seed germination and plant growth. Therefore, using the estimated regression between the soil wetness index and NDVI may not give highly accurate predictions. Obtaining historic weather data from Rawdhat Khuraym will possibly lead to more accurate results compared to the coarse resolution of satellite data.

The NDVI provided a valuable approach for monitoring changes in vegetation cover under arid and semi-arid areas; however, it has some limitations. The first is the coarser resolution (30 m) of Landsat data. It provides a valuable long-term record, but it is inherently limited in its ability to resolve small-scale vegetation patches characteristic of this arid region [69]. The second is the effect of soil background in sparse vegetation areas, where the underlying soil reflectance may contribute significantly to the observed NDVI [70]. The third is using the threshold approach with NDVI, which may result in some uncertainties, especially in arid environments characterized by low and sparse vegetation. In a future study, other vegetation indices (i.e., SAVI, mSAVI, and oSAVI) can be applied to improve result accuracy and minimize uncertainties.

## Conclusions

The present study provides an updated status of the plant diversity of Rawdhat Khuraym and shows that it is a hotspot zone within a desert ecosystem, with a high plant diversity of 89 recorded plant species (57.3% annuals and 42.4% perennials). Dominant plant communities confirmed the three distinctive parts in Rawdhat Khuraym, each with its dominant and important species and associated soil parameters. Our findings revealed a shift in vegetation dominance from sensitive annuals to disturbance-tolerant species, suggesting a shift in vegetation pattern with respect to time and space. The spatial and temporal vegetation-soil relationship supports our field-based assumption that plant communities are shifting from sensitive annuals to disturbance-tolerant xerophytic species like *Rhazia stricta*, *Capparis spinosa*, and *Ziziphus nummularia*. These vegetation patterns are in agreement with the reported ecological responses in arid and semiarid regions, where harsh environmental conditions and anthropogenic activities favor drought-adapted, disturbance-tolerant species [71]. Furthermore, moisture availability was shown to be a more crucial factor controlling the vegetation dynamics and composition compared to temperature, where the plants growing in harsh arid conditions can tolerate the high temperature but not sustain water limitation. During the last five years, a vegetation recovery has been observed that could be attributed to increased rainfall and the establishment of a protected area that prohibits grazing.

The results are directly applicable to the conservationa and restoration of this culturally and ecologically important rawdah. For example, the current floristic composition can inform which species are missing for restoration purposes. Moreover, the found relationship between soil parameters and plant communities can specify the most suitable species to plant depending on the soil type and, therefore, greatly enhance the establishment rate of the newly introduced species and contribute to improved restoration approaches. Lastly, if native herbivores are to be reintroduced, the species composition can be used for habitat suitability in feasibility studies. To incorporate climate change impacts on the ecological dynamics and restoration activities, additional research is needed to provide more in-depth information on the small-scale predicted impacts and to use an ensemble model for vegetation indices to account for uncertainties in the predictions of vegetation trajectories. 

## Supplementary Information

Supplementary Material 1: Table S1: The coordinates and altitude of the studied plots within Rawdhat Khuraym, Saudi Arabia. Table S2: The comparative floristic composition of Rawdhat Khuraym of the previously published work and the current study. : Table S3: The Pearson Correlation Coefficient (r) of the soil variables and the dominant and important plants of the identified plant communities within Rawdhat Khuraym, Saudi Arabia, during the winter-spring season. Table S4: The Pearson Correlation Coefficient (r) of the soil variables and the dominant and important plants of the identified plant communities within Rawdhat Khuraym, Saudi Arabia, during the summer-fall season. Fig. S1: Floristic composition of the studied sites within Rawdhat Khuraym, Saudi Arabia. Fig. S2: Hierarchical cluster analysis of the studied sites of Rawdhat Khuraym, Saudi Arabia, during the winter-spring and summer-fall seasons. Fig. S3: Sentinel 2 false color composite image and extracted vegetation cover during the period from 1986 to 1995. Fig. S4: Sentinel 2 false color composite image and extracted vegetation cover during the period from 1996 to 2005. Fig. S5: Sentinel 2 false color composite image and extracted vegetation cover during the period from 2006 to 2015. Fig. S6: Sentinel 2 false color composite image and extracted vegetation cover during the period from 2016 to 2025. Fig. S7: NDVI values and monthly precipitation in the different studied parts of Rawdhat Khuraym from 1986 to 2025 at two months' time lag.

## Data Availability

All data are available within the manuscript and the supplementary materials.
