# Supplementary material for: Plant diversity, seasonal dynamics, and vegetation-soil relationship of Rawdhat Khuraym, Saudi Arabia: a biodiversity hotspot region within a hyper-arid region
Source: BMC Plant Biol. 2026 Jun 8;26:1211. doi: 10.1186/s12870-026-09209-y (PMC13374360; doi:10.1186/s12870-026-09209-y)
Supplement: Supplementary file 1 — Supplementary Material 1: Table S1: The coordinates and altitude of the studied plots within Rawdhat Khuraym, Saudi Arabia. Table S2: The comparative floristic composition of Rawdhat Khuraym of the previously published work and the current study. : Table S3: The Pearson Correlation Coefficient (r) of the soil variables and the dominant and important plants of the identified plant communities within Rawdhat Khuraym, Saudi Arabia, during the winter-spring season. Table S4: The Pearson Correlation Coefficient (r) of the soil variables and the dominant and important plants of the identified plant communities within Rawdhat Khuraym, Saudi Arabia, during the summer-fall season. Fig. S1: Floristic composition of the studied sites within Rawdhat Khuraym, Saudi Arabia. Fig. S2: Hierarchical cluster analysis of the studied sites of Rawdhat Khuraym, Saudi Arabia, during the winter-spring and summer-fall seasons. Fig. S3: Sentinel 2 false color composite image and extracted vegetation cover during the period from 1986 to 1995. Fig. S4: Sentinel 2 false color composite image and extracted vegetation cover during the period from 1996 to 2005. Fig. S5: Sentinel 2 false color composite image and extracted vegetation cover during the period from 2006 to 2015. Fig. S6: Sentinel 2 false color composite image and extracted vegetation cover during the period from 2016 to 2025. Fig. S7: NDVI values and monthly precipitation in the different studied parts of Rawdhat Khuraym from 1986 to 2025 at two months' time lag. [file 12870_2026_9209_MOESM1_ESM.docx]

**Supplementary Materials**

**Table S1** The coordinates and altitude of the studied plots within Rawdhat Khuraym, Saudi Arabia.

| **Ecological Zones** | **Plots with Quadrat** | **Longitude** | **Latitude** | **Altitude** |
| --- | --- | --- | --- | --- |
| Nothern Part | N1Q1 | 25.431080 | 47.238710 | 523 |
|  | N1Q2 | 25.432370 | 47.237750 | 549 |
|  | N2Q1 | 25.428005 | 47.242352 | 555 |
|  | N2Q2 | 25.427154 | 47.241686 | 554 |
|  | N3Q1 | 25.427844 | 47.238690 | 556 |
|  | N3Q2 | 25.429367 | 47.236812 | 554 |
|  | N4Q1 | 25.425502 | 47.243527 | 554 |
|  | N4Q2 | 25.426525 | 47.241911 | 555 |
|  | N5Q1 | 25.422152 | 47.245042 | 554 |
|  | N5Q2 | 25.422493 | 47.243147 | 555 |
|  | N6Q1 | 25.419730 | 47.252774 | 555 |
|  | N6Q2 | 25.419380 | 47.251224 | 554 |
|  | N7Q1 | 25.412248 | 47.251927 | 556 |
|  | N7Q2 | 25.413362 | 47.251291 | 556 |
| Central Part | C1Q1 | 25.407187 | 47.258483 | 554 |
|  | C1Q2 | 25.407187 | 47.261983 | 556 |
|  | C2Q1 | 25.386486 | 47.273766 | 552 |
|  | C2Q2 | 25.389242 | 47.273744 | 556 |
|  | C3Q1 | 25.384476 | 47.277411 | 553 |
|  | C3Q2 | 25.385284 | 47.279241 | 552 |
|  | C4Q1 | 25.396710 | 47.260620 | 556 |
|  | C4Q2 | 25.396260 | 47.259060 | 556 |
| Southern Part | S1Q1 | 25.325404 | 47.333150 | 552 |
|  | S1Q2 | 25.326343 | 47.334475 | 551 |
|  | S2Q1 | 25.340433 | 47.327777 | 546 |
|  | S2Q2 | 25.341379 | 47.326090 | 547 |
|  | S3Q1 | 25.331471 | 47.324783 | 546 |
|  | S3Q2 | 25.332971 | 47.324992 | 546 |
|  | S4Q1 | 25.345830 | 47.317479 | 546 |
|  | S4Q2 | 25.347082 | 47.316645 | 546 |
|  | S5Q1 | 25.361299 | 47.305270 | 551 |
|  | S5Q2 | 25.362267 | 47.308385 | 550 |
|  | S6Q1 | 25.369700 | 47.294300 | 546 |
|  | S6Q2 | 25.372790 | 47.297160 | 548 |
|  | S7Q1 | 25.356520 | 47.295700 | 548 |
|  | S7Q2 | 25.356670 | 47.297370 | 547 |
|  | S8Q1 | 25.351760 | 47.288750 | 549 |
|  | S8Q2 | 25.353390 | 47.290540 | 553 |
|  | S9Q1 | 25.345335 | 47.302116 | 552 |
|  | S9Q2 | 25.344622 | 47.300826 | 551 |
|  | S10 Q1 | 25.348130 | 47.303890 | 548 |
|  | S10 Q2 | 25.349407 | 47.304965 | 549 |

**Table S2** The comparative floristic composition of Rawdhat Khuraym of the previously published work and the current study.

| **Species List** | **Family** | **El-Din et. al (1994)** | **Al-Farhan (2001)** | **Shalabi and Aljaloud (2003)** | **Current Study** | **Growth Form** | **Life Form** | **Drought Resistant** | **Salt Resistant** | **Flood Tolerant** | **Ecological Significance** |
| --- | --- | --- | --- | --- | --- | --- | --- | --- | --- | --- | --- |
| *Acacia salicina* Lindl. | Fabaceae | 0 | 0 | 0 | 1 | Tree | Phanerophyte | High | Moderate | High | N-Fixer, Fodder, Honey production, Ornamental, Weeper Plant |
| *Achillea fragrantissima* (Forssk.) Sch.Bip. | Asteraceae | 1 | 1 | 1 | 1 | Sub-Shrub | Chamaephyte | High | Moderate | Low | Traditional Medicine. |
| *Aegialina pumila* (Lam.) Quintanar & Barberá | Poaceae | 0 | 0 | 1 | 0 | Annual herb | Therophyte | Moderate | High | Moderate | Component of grassland ecosystem |
| *Aerva javanica* (Burm.f.) Juss. ex Schult. | Amaranthaceae | 0 | 0 | 0 | 1 | Sub-Shrub | Chamaephyte | High | Moderate | Low | Dune stabilization. Traditional medicine |
| *Aizoon canariense* L. | Aizoaceae | 0 | 1 | 0 | 0 | Perennial herb | Therophyte | High | Low | Low | Reduces soil erosion, allelopathic |
| *Aizoon hispanicum* L. | Aizoaceae | 0 | 1 | 0 | 0 | Annual herb | Therophyte | High | High | Low | Pioneer halophyte |
| *Ammi majus* L. | Apiaceae | 0 | 1 | 0 | 0 | Annual herb | Therophyte | Less | Low | Low | Nectar, a seed-eating bird, invasive. |
| *Andrachne telephioides* L. | Phyllanthaceae | 0 | 1 | 1 | 0 | Sub-Shrub | Chamaephyte | Very High | Moderate | Low | NA |
| *Anisosciadium lanatum* Boiss. | Apiaceae | 0 | 0 | 1 | 1 | Annual herb | Therophyte | High | Moderate | Low | Edible. Medicinal. Pollinator. |
| *Anthemis deserti* Boiss. | Asteraceae | 0 | 1 | 1 | 1 | Annual herb | Therophyte | High | Moderate | Low | Aromatic. Medicinal. Pollinator attracter |
| *Anthemis pseudocotula* Boiss. | Asteraceae | 0 | 1 | 0 | 0 | Annual herb | Therophyte | High | Moderate | Low | Essential oil. Medicinal. Pollinator |
| *Anthemis zoharyana* Eig | Asteraceae | 0 | 1 | 0 | 0 | Annual herb | Therophyte | High | Moderate | Low | Potential Pollinator. Rare |
| *Anvillea garcinii* (Burm.f.) DC. | Asteraceae | 0 | 0 | 1 | 0 | Sub-Shrub | Chamaephyte | Very High | Moderate | Low | Traditional medicine. |
| *Arnebia decumbens* (Sieber ex Lehm.) A.DC. | Boraginaceae | 0 | 1 | 0 | 0 | Annual herb | Therophyte | High | Moderate | Low | Pollinator. Fodder and Medicinal uses. |
| *Arnebia hispidissima*(Sieber ex Lehm.) A.DC. | Boraginaceae | 0 | 1 | 1 | 0 | Annual herb | Therophyte | High | Moderate | Low | Medicinal. Pollen supporter |
| *Artemisia scoparia* Waldst. & Kit. | Asteraceae | 0 | 0 | 1 | 0 | Perennial herb | Hemicryptophyte | High | Moderate | Low | Traditional medicine. Aromatic. |
| *Asphodelus tenuifolius* Cav. | Liliaceae | 0 | 1 | 0 | 1 | Annual herb | Geophtye | High | Moderate | Moderate | Weed .Traditional medicine. Ornamental. |
| *Asphodelus fistulosus* L. | Asphodelaceae | 0 | 0 | 1 | 0 | Perennial herb | Geophtye | High | High | Low | Invasive weed in Australia. |
| *Astragalus crenatus* Schult. | Fabaceae | 0 | 1 | 0 | 1 | Annual herb | Therophyte | High | Moderate | Low | Nitrogen Fixing |
| *Astragalus schimperi* Boiss. | Fabaceae | 0 | 1 | 0 | 0 | Annual herb | Therophyte | High | Moderate | Low | Nitrogen Fixing |
| *Astragalus sieberi* DC. | Fabaceae | 0 | 1 | 0 | 0 | Sub-Shrub | Chamaephyte | High | Moderate | Low | Nitrogen Fixing |
| *Astragalus spinosus* (Forssk.) Muschl. | Fabaceae | 1 | 1 | 1 | 1 | Shrub | Chamaephyte | High | Moderate | Low | Nitrogen Fixing |
| *Astragalus tribuloides* Delile | Fabaceae | 0 | 1 | 0 | 0 | Annual herb | Therophyte | High | Moderate | Low | Nitrogen Fixing |
| *Atractylis cancellata* L. | Asteraceae | 0 | 0 | 0 | 1 | Annual herb | Therophyte | High | Low | Low | NA |
| *Atractylis carduus* (Forssk.) C.Chr. | Asteraceae | 0 | 1 | 1 | 0 | Perennial herb | Chamaephyte | High | Moderate | Low | Xeropytic Thistle |
| *Avena fatua* L. | Poaceae | 0 | 0 | 0 | 1 | Annual grass | Therophyte | Moderate | Low | Low | Weed, forage, seeds for granivorous birds |
| *Bassia muricata* (L.) Asch. | Amaranthaceae | 0 | 1 | 1 | 0 | Annual herb | Therophyte | High | High | Low | NA |
| *Beta vulgaris* L. | Amaranthaceae |  | 1 | 0 |  | Biennial herb | Hemicryptophyte | Moderate | Moderate | Low | Fodder, Ruderal vegetation |
| *Blepharis attenuata* Naper | Acanthaceae | 0 | 0 | 0 | 1 | Perennial herb | Chamaephyte | High | Moderate | Low | Xerophytic Spiny. Pharmacological |
| *Bromus pulchellus* Fig. & De Not. | Poaceae |  | 1 | 0 | 0 | Annual grass | Therophyte | High | Low | Low | Seasonal ephemeral communities |
| *Bromus tectorum* L. | Poaceae | 0 | 0 | 0 | 1 | Annual grass | Therophyte | High | Low | Low | Increase Fire Frequency. Invasive |
| *Cakile arabica* Velen. & Bornm. | Brassicaceae | 0 | 1 | 0 | 1 | Annual herb | Therophyte | High | High | Moderate | Coastal Stabilizer |
| *Calendula arvensis* L. | Asteraceae | 0 | 1 | 1 | 1 | Annual herb | Therophyte | Moderate | Low | Low | Attracts pollinators.Edible.Medicinal. |
| *Calendula tripterocarpa Rupr.* | Asteraceae |  | 1 | 0 | 0 | Annual herb | Therophyte | High | Low | Low | Seasonal nectar, Folk medicine |
| *Calotropis procera* (Aiton) Aiton f. | Asclepiadaceae | 1 | 1 | 1 | 1 | Shrub | Phanerophyte | Very High | High | Moderate | Invasive pioneer. Toxic. Medicinal. Fiber. Rubber source. |
| *Capparis decidua* (Forssk.) Edgew. | Capparaceae | 1 | 1 | 1 | 1 | Tree | Phanerophyte | Very High | High | Low | Desert Stabilizer. Folk medicine. |
| *Capparis spinosa* L. | Capparaceae | 1 | 1 | 1 | 1 | Shrub | Phanerophyte | Very High | Very High | Low | Soil stabilizer. Ornamental. Medicinal. Edible |
| *Carthamus oxyacanthus* M.Bieb. | Asteraceae | 1 | 0 | 0 | 1 | Annual herb | Therophyte | Very High | Moderate | Low | Disease Resistant. Allelopathic. Medicinal |
| *Caylusea hexagyna* (Forssk.) M.L.Green | Resedaceae | 0 | 1 | 1 | 0 | Annual herb | Therophyte | Very High | Moderate | Low | Edible Green. Fodder. Medicinal Use |
| *Cenchrus ciliaris* L. | Poaceae | 0 | 1 | 1 | 1 | Annual grass | Hemicryptophyte | Very High | Moderate | Moderate | Promotes Fire, Forage. Soil stabilizer. Outcompete natives |
| *Cenchrus divisus* (J.F.Gmel.) Verloove, Govaerts & Buttler | Poaceae | 1 | 1 | 1 | 0 | Annual grass | Geophtye | Very High | Moderate | Low | Rhizomatous, Forage |
| *Centaurea sinaica* DC. | Asteraceae | 0 | 0 | 0 | 1 | Sub-Shrub | Chamaephyte | High | Moderate | Low | Medicinal potential. |
| *Centropodia forskaolii* (Vahl) Cope | Poaceae | 0 | 1 | 0 | 0 | Grass perennial | Hemicryptophyte | Very High | Moderate | Low | Key binding grass. Stabilizes sand dunes. |
| *Centropodia fragilis* (Guinet & Sauvage) Cope | Poaceae | 0 | 0 | 1 | 0 | Grass perennial | Hemicryptophyte | Very High | Moderate | Low | Pioneer stabilizer. |
| *Chenopodiastrum murale* (L.) S.Fuentes, Uotila & Borsch | Amaranthaceae | 0 | 1 | 1 | 0 | Annual herb | Therophyte | Moderate | Moderate | Low | Nutrient accumulator. weed. allelopthic |
| *Chrozophora oblongifolia* (Delile) A.Juss. ex Spreng. | Euphorbiaceae | 1 | 0 | 1 | 0 | Sub-Shrub | Chamaephyte | High | Moderate | Low | Traditional medicine. Potential Toxic. Supports arid shrubland diversity. |
| *Chrozophora tinctoria* (L.) Raf. | Euphorbiaceae | 0 | 1 | 1 | 1 | Annual herb | Therophyte | Moderate | Low | Low | Dye Source. Traditional medicine. |
| *Citrullus colocynthis* (L.) Schrad. | Cucurbitaceae | 1 | 1 | 1 | 1 | Perennial herb | Geophtye | Very High | High | Low | Medicinal. Cooked seeds edible |
| *Cleome amblyocarpa* Barratte & Murb. | Capparaceae | 0 | 1 | 1 | 1 | Annual herb | Therophyte | High | Moderate | Low | Traditional Medicine. Glandular herb |
| *Cleome arabica* L. | Cleomaceae | 1 | 0 |  | 0 | Perennial herb | Therophyte | High | Moderate | Low | Traditional Medicine |
| *Coincya tournefortii* (Gouan) Alcaraz, T.E.Díaz | Brassicaceae | 0 | 1 | 1 | 1 | Annual herb | Therophyte | High | Moderate | Low | Allelopathic |
| *Convolvulus cephalopodus* Boiss. | Convolvulaceae | 0 | 0 | 0 | 1 | Shrub | Chamaephyte | High | Moderate | Low | resilient to grazing and trampling |
| *Convolvulus fatmensis* Kunze | Convolvulaceae | 0 | 1 | 0 | 0 | Sub-Shrub | Hemicryptophyte | High | Low | Low | Soil surface stabilizer, |
| *Convolvulus oxyphyllus* Boiss. | Convolvulaceae | 1 | 1 | 1 | 1 | Sub-Shrub | Chamaephyte | High | Moderate | Low | Defense against overgrazing, desert biodiversity, and pollinator |
| *Convolvulus pilosellifolius* Desr. | Convolvulaceae | 0 | 1 | 1 | 1 | Sub-Shrub | Chamaephyte | High | Low | Low | Grazing Plant |
| *Convolvulus prostratus* Forssk. | Convolvulaceae | 0 | 1 | 1 | 0 | Sub-Shrub | Chamaephyte | Very High | Moderate | Low | Traditional Medicine |
| *Cucumis prophetarum* L. | Cucurbitaceae | 0 | 1 | 0 | 0 | Perennial herb | Geophtye | High | Moderate | Low | Stabilizing sand soil during wet pulses. |
| *Cutandia memphitica* (Spreng.) K.Richt. | Poaceae | 0 | 1 | 1 | 0 | Annual grass | Therophyte | High | High | Low | Grazed in rangeland |
| *Cynodon dactylon* (L.) Ps. | Poaceae | 1 | 1 | 1 | 1 | Annual grass | Hemicryptophyte | Very High | High | Moderate | Invasive |
| *Cyperus conglomeratus* Rottb. | Cyperaceae | 0 | 0 | 1 | 0 | Annual grass | Geophtye | Very High | High | Moderate | Forage with Medicinal Values |
| *Delphinium pubescens* DC. | Ranunculaceae | 0 | 1 | 0 | 0 | Annual herb | Therophyte | Moderate | Low | Low | Nectar and pollen, ephemeral biodiversity |
| *Dichanthium foveolatum* (Delile) Roberty | Poaceae | 0 | 0 | 1 | 0 | Annual grass | Hemicryptophyte | High | Moderate | Low | Livestock forage |
| *Dipcadi erythraeum* Webb & Berthel. | Asparagaceae | 0 | 0 | 1 | 0 | Annual Herb | Geophtye | Very High | Moderate | Low | Culinary and Medicine |
| *Diplotaxis acris* (Forssk.) Boiss. | Brassicaceae | 0 | 0 | 1 | 0 | Annual herb | Therophyte | High | Moderate | Low | Edible Green |
| *Diplotaxis harra* (Forssk.) Boiss. | Brassicaceae | 0 | 0 | 1 | 0 | Annual herb | Therophyte | High | Moderate | Low | Edible Green and Medicinal |
| *Echium arabicum* R. Mill ( Kahal) | Brassicaceae | 0 | 1 | 0 | 0 | Perennial herb | Hemicryptophyte | High | Moderate | Low | Nectar source. Stabilizes Wadi banks |
| *Echium rauwolfii* Delile | Boraginaceae | 0 | 0 | 0 | 1 | Annual herb | Therophyte | High | Moderate | Low | Pastoral Forage, Pollinator attractor |
| *Eleusine indica* (L.) Gaertn. | Poaceae | 0 | 0 | 0 | 1 | Annual grass | Therophyte | Moderate | Moderate | Moderate | Famine Food/ Agricultural Weed |
| *Ephedra ciliata* Fisch. & C.A.Mey. | Ephedraceae | 1 | 0 | 1 | 1 | Shrub | Phanerophyte | Very High | Moderate | Low | Medicinal |
| *Eragrostis barrelieri* Daveau | Poaceae | 0 | 1 | 0 | 0 | Annual grass | Therophyte | Moderate | Low | Low | Forage/Soil Stabilizer |
| *Eremobium aegyptiacum* (Spreng.) Asch. ex Boiss. | Brassicaceae | 0 | 1 | 0 | 0 | Annual herb | Therophyte | Very High | Moderate | Low | Sand stabilizer of Empty Quarter |
| *Erigeron bonariensis* L. | Asteraceae | 0 | 0 | 0 | 1 | Annual herb | Therophyte | High | Moderate | Low | Invader of overgrazed rangeland |
| *Erodium touchyanum* Delile ex Godr. | Geraniaceae | 0 | 1 | 1 | 0 | Annual herb | Therophyte | High | Moderate | Low | Post rain stablizer |
| *Erodium laciniatum* (Cav.) Willd. | Geraniaceae | 0 | 1 | 1 | 1 | Annual herb | Therophyte | High | Moderate | Low | Soil Stabilizer via seed burial |
| *Eruca sativa* Mill. | Brassicaceae | 0 | 1 | 1 | 0 | Annual herb | Therophyte | Moderate | Moderate | Low | Edible Green, Pest Defense |
| *Erucaria hispanica* (L.) Druce | Brassicaceae | 0 | 1 | 1 | 0 | Biennial herb | Therophyte | High | Moderate | Low | Edible Wild Green |
| *Anastatica hierachuntica* Crantz | Brassicaceae | 0 | 1 | 1 | 0 | Annual herb | Therophyte | High | Moderate | Low | Rain dispersal strategist, post-flood wadi colonizer, folk medicine |
| *Euphorbia dracunculoides* Lam. | Euphorbiaceae | 0 | 1 | 1 | 0 | Annual herb | Therophyte | High | Moderate | Low | Medicinal |
| *Euphorbia granulata* Forssk. | Euphorbiaceae | 0 | 1 | 1 | 1 | Annual herb | Therophyte | Very High | Moderate | Low | Traditional Medicine (Toxic latex) |
| *Fagonia indica* Burm.f. | Zygophyllaceae | 1 | 1 | 1 | 1 | Sub-Shrub | Chamaephyte | Very High | High | Low | Medicinal |
| *Farsetia aegyptia* Turra | Brassicaceae | 0 | 1 | 1 | 1 | Shrub | Chamaephyte | Very High | High | Low | Traditional Medicine |
| *Farsetia longisiliqua* Decne. | Brassicaceae | 0 | 1 | 0 | 0 | Sub-Shrub | Chamaephyte | High | Moderate | Low | Stability of Shrubland Habitat |
| *Farsetia stylosa* R.Br. | Brassicaceae | 0 | 1 | 0 | 0 | Sub-Shrub | Chamaephyte | Very High | Moderate | Low | Forage |
| *Filago desertorum* Pomel | Asteraceae | 0 | 1 | 1 | 1 | Annual herb | Therophyte | Very High | Low | Low | Stabilizes silt deposits |
| *Flaveria trinervia* (Spreng.) C.Mohr | Asteraceae | 0 | 1 | 0 | 0 | Annual herb | Therophyte | Low | High | High | Invasive, minro nectar/pollen resources, Rapid colonizer of disturbed wetlands. |
| *Forsskaolea tenacissima* L. | Urticaceae | 0 | 0 | 0 | 1 | Perennial herb | Chamaephyte | Very High | Moderate | Low | Palatable livestock forage |
| *Galium ceratopodum* Boiss. | Rubiaceae | 0 | 0 | 1 | 0 | Annual herb | Therophyte | Very High | Low | Low | Soil Stabilizer in Hyper-arid zones |
| *Gastrocotyle hispida* (Forssk.) Bunge | Boraginaceae | 0 | 1 | 0 | 0 | Perennial herb | Hemicryptophyte | High | Low | Low | Early successional vegetation, nectar/pollen for pollinators, and forage. |
| *Gypsophila capillaris* (Forssk.) C.Chr. | Caryophyllaceae | 0 | 1 | 1 | 1 | Perennial herb | Hemicryptophyte | Very High | Low | Low | Contributes to floristic diversity, indicating stability |
| *Haloxylon salicornicum* (Moq.) Bunge ex Boiss. | Amaranthaceae | 0 | 1 | 1 | 0 | Shrub | Chamaephyte | Very High | High | Low | Forage, microhabitat for understory ephemerals, and carbon sequestration |
| *Haplophyllum tuberculatum* (Forssk.) A.Juss. | Rutaceae | 1 | 1 | 1 | 1 | Shrub | Hemicryptophyte | High | Moderate | Low | Pioneer in post disturbance, medicinal compounds |
| *Heliotropium angiospermum* Murray | Boraginaceae | 0 | 0 | 1 | 0 | Sub-Shrub | Therophyte | High | Moderate | Moderate | Sand Stabilizer, Bee attractor, fast-growing ground cover. |
| *Heliotropium bacciferum* Forssk. | Boraginaceae | 0 | 1 | 0 | 0 | Sub-Shrub | Chamaephyte | High | Moderate | Low | Livestock forage. Medicinal. Edible |
| *Heliotropium digynum* (Forssk.) Asch. ex C.Chr. | Boraginaceae | 0 | 1 | 1 | 0 | Sub-Shrub | Chamaephyte | Very High | Low | Low | Sand stabilization and post-rain ephemeral |
| *Heliotropium ramosissimum* (Lehm.) Sieber ex DC. | Boraginaceae | 1 | 1 | 1 | 1 | Sub-Shrub | Chamaephyte | Very High | Moderate | Low | Higher winter richness, soil stabilization, and seed bank. |
| *Herniaria hirsuta* L. | Caryophyllaceae | 0 | 1 | 1 | 0 | Annual herb | Therophyte | High | Low | Low | Form dense mats, enhancing post-rain biodiversity and soil stability. |
| *Hordeum murinum* L. | Poaceae | 0 | 0 | 1 | 1 | Annual grass | Therophyte | High | Moderate | Low | High-biomass livestock fodder |
| *Horwoodia dicksoniae* Turrill | Brassicaceae | 0 | 1 | 1 | 0 | Annual herb | Therophyte | Very High | Low | Low | Indicator of stable desert plains, aromatic, biodiversity hotspot. |
| *Hypecoum pendulum* L. | Papaveraceae | 0 | 1 |  | 0 | Annual herb | Therophyte | Very High | Low | Low | Indicator of seasonal productivity and stability |
| *Ifloga spicata* (Forssk.) Sch.Bip. | Asteraceae | 0 | 1 | 1 | 0 | Annual herb | Therophyte | Very High | Low | Low | stabilizing sand dunes, indicating erratic rainfall events. |
| *Koelpinia linearis* Pall. | Asteraceae | 0 | 1 | 1 | 0 | Annual herb | Therophyte | Very High | Low | Low | Sand Stabilizer in hyper-arid communities |
| *Lactuca serriola* L. | Asteraceae | 0 | 0 | 0 | 1 | Biennial herb | Therophyte | High | Low | Low | Weed in crops, forage for wildlife, and are toxic to livestock. Indicates disturbed nutrient-rich sites. |
| *Lappula spinocarpos* (Forssk.) Asch. ex Kuntze | Boraginaceae | 0 | 1 | 1 | 0 | Annual herb | Therophyte | Very High | Low | Low | Aiding seed dispersal via the spiny nutlet |
| *Lasiurus scindicus* Henrard | Poaceae | 1 | 1 | 1 | 1 | Annual grass | Geophtye | Very High | Moderate | Low | Forage, Sand binder, supports biodiversity |
| *Launaea angustifolia* (Desf.) Kuntze | Asteraceae | 0 | 0 | 0 | 1 | Annual herb | Therophyte | Very High | Low | Low | Support ephemeral biodiversity in arid ecosystems |
| *Launaea capitata* (Spreng.) Dandy | Asteraceae | 0 | 1 | 1 | 1 | Annual herb | Therophyte | Very High | Low | Low | Stabilizing substrate, indicating seasonal moisture in hyperarid regions. |
| *Launaea nudicaulis* (L.) Hook.f. | Asteraceae | 0 | 1 | 0 | 1 | Perennial herb | Chamaephyte | Very High | Moderate | Low | Supporting biodiversity and traditional use |
| *Launaea procumbens* (Roxb.) Ramayya & Rajagopal | Asteraceae | 0 | 0 | 0 | 1 | Perennial herb | Chamaephyte | High | Moderate | Low | Supporting biodiversity and traditional use |
| *Lappula spinocarpos* (Forssk.) Asch. ex Kuntze | Boraginaceae | 0 | 1 | 0 | 0 | Annual herb | Therophyte | Moderate | Low | Low | Ruderal Species in Disturbed Areas |
| *Leontodon laciniatus* (Bertol.) Widder ex Bornm. | Asteraceae | 0 | 1 | 0 | 0 | Annual herb | Therophyte | High | Low | Low | Seasonal cover in the ephemeral desert supports pollinators, |
| *Lepidium aucheri* Boiss. | Brassicaceae | 0 | 1 | 1 | 0 | Perennial herb | Hemicryptophyte | High | Moderate | Low | Floristic Diversity |
| *Leptaleum filifolium* (Willd.) DC. | Brassicaceae | 0 | 1 | 0 | 0 | Annual herb | Therophyte | High | Moderate | Low | Rapid colonizer of post-flood wadi beds, ephemeral biodiversity |
| *Limeum humile* Forssk. | Limeaceae | 0 | 0 | 1 | 0 | Annual herb | Therophyte | High | Moderate | Low | contributes to ground cover for desert dunes and wadis |
| *Lolium multiflorum* Lam. | Poaceae | 0 | 0 | 1 | 0 | Annual grass | Hemicryptophyte | Moderate | Moderate | Low | Forage, soil stabilization, erosion control, but has invasive potential |
| *Lolium rigidum* Gaudin | Poaceae | 0 | 1 | 1 | 1 | Annual Grass | Therophyte | Moderate | Moderate | Low | Forage potential but invasive risk as a weed in disturbed rangelands |
| *Lycium shawii* Roem. & Schult. | Solanaceae | 1 | 1 | 1 | 1 | Shrub | Phanerophyte | High | High | Low | Key browse for camels, traditional medicine |
| *Malva ludwigii* (L.) Soldano, Banfi & Galasso | Malvaceae |  | 1 | 1 |  | Annual herb | Therophyte | High | Moderate | Low | Edible, ruderal pioneer, nitrogen cycle |
| *Malva neglecta* Wallr. | Malvaceae | 0 | 1 | 0 | 1 | Perennial herb | Therophyte | Moderate | Moderate | Low | Pollinator, Edible, ruderal recolonizer of bare soil |
| *Malva parviflora* L. | Malvaceae | 0 | 1 | 1 | 1 | Annual herb | Therophyte | Moderate | Moderate | Low | Attracts pollinators, Weed. |
| *Malva verticillata* L. | Malvaceae | 0 | 1 | 0 | 0 | Annual herb | Therophyte | Moderate | Low | Low | Dense stands in disturbed sites attract pollinators, invasive tendencies |
| *Matthiola longipetala* (Vent.) DC. | Brassicaceae | 0 | 1 | 0 | 0 | Perennial herb | Therophyte | Moderate | Low | Low | Night-blooming fragrance attracts moths and pollinators, forming clumps in disturbed sites. |
| *Matricaria aurea* (Loefl.) Sch.Bip. | Asteraceae | 0 | 1 | 1 | 0 | Annual herb | Therophyte | Moderate | Low | Low | Herbal tea from flower buds, common weed, seasonal ground cover. |
| *Medicago laciniata* (L.) Mill. | Fabaceae | 0 | 1 | 1 | 1 | Annual herb | Therophyte | Very High | Moderate | Low | Forage crop, Nitrogen fixer. |
| *Melilotus indicus* (L.) All. | Fabaceae | 0 | 1 | 0 | 0 | Perennial herb | Therophyte | Moderate | Low | Low | Pollinator attracter, edible, competitive ruderal, and invasive tendencies. |
| *Moltkiopsis ciliata* (Forssk.) I.M.Johnst. | Boraginaceae | 0 | 1 | 1 | 1 | Sub-Shrub | Chamaephyte | High | Moderate | Low | Forage for camel, Sand stabilizer, shows ozone resistance in polluted sites. |
| *Mutarda arvensis* (L.) D.A.German | Brassicaceae | 0 | 1 | 0 | 0 | Annual herb | Therophyte | Moderate | Low | Low | Weed for crops, seed for birds, pollen for bees, seedbank |
| *Neotorularia torulosa* (Desf.) Hedge & J.Léonard | Brassicaceae | 0 | 1 | 0 | 0 | Annual herb | Therophyte | High | Low | Low | Arid biodiveristy |
| *Neurada procumbens* L. | Neuradaceae | 0 | 1 | 0 | 1 | Annual herb | Therophyte | Very High | Low | Low | Sand stabilization |
| *Notoceras bicorne* (Aiton) Amo | Brassicaceae | 0 | 1 | 0 | 1 | Annual herb | Therophyte | High | Moderate | Low | Honey nectar plant. |
| *Oligomeris linifolia* (Vahl ex Hornem.) J.F.Macbr. | Resedaceae | 0 | 1 | 0 | 0 | Annual herb | Therophyte | High | High | Low | Stabilizing soil post-rain, Pioneer in disturbed habitats. |
| *Orobanche pubescens* d'Urv. | Orobanchaceae | 0 | 0 | 0 | 1 | Annual herb | Therophyte | Moderate | Low | Low | Traditional medicine |
| *Otoglyphis factorovskyi* (Warb. & Eig) Oberpr. & Vogt | Asteraceae | 0 | 0 | 0 | 1 | Annual herb | Therophyte | High | Moderate | Moderate | Edible bulb, Traditional medicine. |
| *Panicum repens* L. | Poaceae | 0 | 0 | 1 | 0 | Annual grass | Hemicryptophyte | Moderate | Moderate | High | Invasive. |
| *Panicum turgidum* Forssk. | Poaceae | 1 | 1 | 1 | 0 | Annual grass | Hemicryptophyte | High | High | Low | Forage plant |
| *Papaver rhoeas* L. | Papaveraceae | 0 | 1 | 0 | 0 | Annual herb | Therophyte | Moderate | Low | Low | Nectar/pollen for bees, Ornamental/weedy properties. |
| *Paronychia arabica* (L.) DC. | Caryophyllaceae | 0 | 1 | 0 | 1 | Annual herb | Therophyte | High | Moderate | Low | Ground cover mats. |
| *Phalaris minor* Retz. | Poaceae | 0 | 1 | 1 | 1 | Annaul Grass | Therophyte | Moderate | Moderate | Low | Competitive weed, forage |
| *Peganum harmala* L. | Nitrariaceae | 0 | 1 | 0 | 0 | Sub-Shrub | Hemicryptophyte | High | High | Low | Invasive plant. Traditional medicine. |
| *Picris cyanocarpa* Boiss. | Asteraceae | 0 | 1 | 0 | 1 | Annual herb | Therophyte | High | Moderate | Low | Ephemeral post-rain dominate, nectar resources, achene heteromorphism |
| *Picris babylonica* Hand.-Mazz. | Asteraceae | 0 | 1 | 1 | 0 | Annual herb | Therophyte | High | Moderate | Low | Ephemeral desert communities |
| *Pimpinella puberula* (DC.) Boiss. | Apiaceae | 0 | 0 | 1 | 0 | Annual herb | Therophyte | Moderate | Low | Low | Steppe biodiversity |
| *Plantago amplexicaulis* Cav. | Plantaginaceae | 0 | 1 | 0 | 1 | Annual herb | Therophyte | Very High | Moderate | Low | Desert pulse species, Traditional medicine |
| *Plantago albicans* L. | Plantaginaceae | 0 | 1 | 0 | 0 | Perennial herb | Hemicryptophyte | High | Moderate | Low | Stabilizes dry slopes |
| *Plantago ciliata* Desf. | Plantaginaceae | 0 | 1 | 1 | 1 | Annual herb | Therophyte | High | Moderate | Low | Spring forage for livestock |
| *Plantago coronopus* L. | Plantaginaceae | 0 | 0 | 1 | 0 | Perennial herb | Hemicryptophyte | Moderate | High | Moderate | Stabilizing coastal dunes and salt marshes. Edible. Invasive tendencies. |
| *Plantago ovata* Forssk. | Plantaginaceae | 0 | 1 | 1 | 1 | Annual herb | Therophyte | High | Moderate | Low | Forage for wildlife/ pollinators |
| *Plantago psammophila* Agnew & Chal.-Kabi | Plantaginaceae | 0 | 1 | 0 | 0 | Annual herb | Therophyte | Very High | Low | Very Low | Ephemeral desert flora |
| *Poa annua* L. | Poaceae | 0 | 1 | 0 | 0 | Annual grass | Therophyte | Moderate | Moderate | Moderate | Disturbed turf providing green color |
| *Poa sinaica* Steud. | Poaceae | 0 | 0 | 1 | 0 | Grass perennial | Hemicryptophyte | High | Moderate | Low | Forage based in high altitudes |
| *Polycarpaea repens* (Forssk.) Asch. & Schweinf. | Caryophyllaceae | 0 | 1 | 1 | 1 | Perennial herb | Chamaephyte | Very High | Moderate | Low | Sand dune stabilizer |
| *Polycarpaea robbairea* (Kuntze) Greuter & Burdet | Caryophyllaceae | 0 | 1 | 1 | 0 | Perennial herb | Hemicryptophyte | Very High | Moderate | Low | Microhabitat of Al-Wahbah Crater Slopes |
| *Polygonum argyrocoleon* Steud. ex Kunze | Polygonaceae | 0 | 1 | 1 | 0 | Annual herb | Therophyte | Moderate | High | Moderate | Aggressive colonizer of saline sites, potential weed |
| *Polygonum aviculare* L. | Polygonaceae | 0 | 0 | 0 | 1 | Annual herb | Therophyte | Moderate | Moderate | Moderate | Trampling-resistant pioneer |
| *Polypogon monspeliensis* (L.) Desf. | Polygonaceae | 0 | 1 | 0 | 0 | Annual grass | Therophyte | Low | High | High | Dense stands in disturbed wetlands |
| *Portulaca oleracea* L. | Portulacaceae | 0 | 0 | 1 | 0 | Annual herb | Therophyte | Very High | High | Moderate | Edible, Aggressive competitor |
| *Prosopis farcta* (Banks & Sol.) J.F.Macbr. | Fabaceae | 0 | 0 | 0 | 1 | Sub-Shrub | Geophtye | Very High | Very High | High | Dense clonal stands, invasive potential |
| *Pteranthus dichotomus* Forssk. | Caryophyllaceae | 0 | 1 | 0 | 0 | Annual herb | Therophyte | Very High | Moderate | Low | Pioneer species in mobile sand dune communities |
| *Pulicaria jaubertii* E.Gamal-Eldin | Asteraceae | 0 | 1 | 0 | 0 | Perennial herb | Hemicryptophyte | High | High | Low | Contributes to arid vegetation cover in wadis and plains. |
| *Pulicaria undulata* (Forssk.) C.A.Mey. | Asteraceae | 1 | 1 | 1 | 1 | Sub-Shrub | Chamaephyte | High | Moderate | Low | Aromatic cover in ephemeral desert communities, medicinal values |
| *Reichardia tingitana* (L.) Roth | Asteraceae | 0 | 1 | 0 | 0 | Biennial herb | Hemicryptophyte | High | High | Low | Pioneer species in disturbed coastal and desert communities, allelopathic activities |
| *Reseda alba* L. | Resedaceae | 0 | 1 | 0 | 0 | Perennial herb | Hemicryptophyte | High | Low | Low | Ornamental and nectar for pollinators |
| *Reseda arabica* Boiss. | Resedaceae | 0 | 1 | 1 | 0 | Annual herb | Therophyte | Very High | Moderate | Low | Contributes to ephemeral desert flora |
| *Reseda aucheri* Boiss. | Resedaceae | 0 | 0 | 0 | 1 | Perennial herb | Hemicryptophyte | High | Moderate | Low | Contributes to desert steppe vegetation |
| *Rhanterium epapposum* Oliv. | Asteraceae | 0 | 1 | 1 | 1 | Shrub | Chamaephyte | Very High | High | Low | Dominant species forming an extensive arid shrubland |
| *Rhazya stricta* Decne. | Apocynaceae | 1 | 1 | 1 | 1 | Shrub | Phanerophyte | Very High | High | Low | Dominant shrub making monospecific stands. Stabilizing sand soil. Medicinal. |
| *Rumex dentatus* L. | Polygonaceae | 0 | 0 | 1 | 0 | Annual herb | Therophyte | Moderate | Moderate | Moderate | Aggressive colonizer of cultivated fields and roadsides. Traditional medicines |
| *Rumex spinosus* L. | Polygonaceae | 0 | 1 | 1 | 1 | Annual herb | Therophyte | High | Moderate | Low | Nuisance weed with barbed spiny seeds |
| *Rumex vesicarius* L. | Polygonaceae | 0 | 1 | 1 | 0 | Annual herb | Therophyte | Moderate | Moderate | High | Rapid colonizer of disturbed wetland habitats. Used in traditional medicines |
| *Salvia aegyptiaca* L. | Lamiaceae | 0 | 1 | 1 | 1 | Sub-Shrub | Chamaephyte | High | Moderate | Low | Contributes to arid shrubland diversity. Allelopathic potentials |
| *Salvia spinosa* L. | Lamiaceae | 0 | 0 | 0 | 1 | Sub-Shrub | Hemicryptophyte | High | Moderate | Low | Pollinator resources in spring desert communities |
| *Savignya parviflora* (Delile) Webb | Brassicaceae | 0 | 0 | 1 | 0 | Annual herb | Therophyte | High | Moderate | Low | Herbecous weed |
| *Schimpera arabica* Hochst. & Steud. | Brassicaceae | 0 | 1 | 0 | 0 | Annual herb | Therophyte | Very High | Moderate | Low | Therophyte-dominated ephemeral flora |
| *Schismus arabicus* Nees | Poaceae | 0 | 0 | 0 | 1 | Annual grass | Therophyte | Very High | Moderate | Low | Invasive fire promoter |
| *Schismus barbatus* (L.) Thell. | Poaceae | 0 | 1 | 1 | 1 | Annual grass | Therophyte | High | Moderate | Low | Competittor |
| *Scorpiurus muricatus* L. | Fabaceae | 0 | 0 | 1 | 0 | Annual herb | Therophyte | Moderate | Low | Low | Nitrogen-fixing annual. Ornamental and cover crop |
| *Scorzonera musilii* Velen. | Asteraceae | 0 | 1 | 0 | 0 | Perennial herb | Hemicryptophyte | High | Moderate | Low | Contributes to desert floral diversity |
| *Rostraria cristata* (L.) Tzvelev | Poaceae | 0 | 1 | 0 | 0 | Annual grass | Therophyte | Moderate | Low | Low | Ruderal species in disturbed habitats |
| *Senna alexandrina* Mill. | Fabaceae | 0 | 1 | 0 | 0 | Shrub | Chamaephyte | High | Moderate | Low | Medicinal, improves soil by nitrogen-fixing roots. |
| *Senna italica Mill.* | Fabaceae | 0 | 0 | 1 | 0 | Sub-Shrub | Chamaephyte | High | Moderate | Low | Invasive in the Caribbean islands. Medicinal. |
| *Senecio aegyptius* L. | Asteraceae | 0 | 0 | 1 | 0 | Annual herb | Therophyte | High | Moderate | Low | Contributes to desert flora diversity. Medicinal value |
| *Senecio glaucus* L. | Asteraceae | 0 | 1 | 0 | 0 | Annual herb | Therophyte | High | High | Low | Pioneer species stabilizing mobile dunes |
| *Silene arabica* Boiss. | Caryophyllaceae | 0 | 1 | 0 | 0 | Annual herb | Therophyte | High | Moderate | Low | Prominent spring flower of wet seasons. Ornamental |
| *Silene conoidea* L. | Caryophyllaceae | 0 | 1 | 0 | 0 | Annual herb | Therophyte | Moderate | Low | Low | Weed in disturbed areas. |
| *Silene villosa* Forssk. | Caryophyllaceae | 0 | 1 | 1 | 0 | Annual herb | Therophyte | Very High | Moderate | Low | Spring ephemeral post-rainfall. |
| *Sisymbrium erysimoides* Desf. | Brassicaceae | 0 | 0 | 1 | 0 | Annual herb | Therophyte | Moderate | Low | Low | Widespread weed of agricultural wheat fields |
| *Sisymbrium irio* L. | Brassicaceae | 0 | 1 | 0 | 1 | Annual herb | Therophyte | Moderate | Moderate | Low | Rapid colonizer of disturbed areas. Edible. |
| *Sisymbrium orientale* L. | Brassicaceae | 0 | 1 | 0 | 0 | Annual herb | Therophyte | Moderate | Low | Very Low | Edible |
| *Sonchus oleraceus* L. | Asteraceae | 0 | 1 | 0 | 0 | Annual herb | Therophyte | Moderate | Moderate | Moderate | Rapid colonizer. Weed capabilities. Edible |
| *Spergularia diandra* (Guss.) Heldr. | Caryophyllaceae | 0 | 1 | 0 | 0 | Annual herb | Therophyte | High | High | Low | Pioneer species in saline/ disturbed sand. |
| *Spergularia flaccida* (Madden) I.M.Turner | Caryophyllaceae | 0 | 1 | 1 | 0 | Annual herb | Therophyte | High | High | Low | Pioneer colonizer of salt-affected soil |
| *Stipagrostis drarii* (Täckh.) De Winter | Poaceae | 1 | 1 | 1 | 0 | Grass perennial | Hemicryptophyte | Very High | Low | Low | Dominant sand dunes |
| *Stipagrostis plumosa* (L.) Munro ex T.Anderson | Poaceae | 1 | 1 | 1 | 0 | Grass perennial | Hemicryptophyte | Very High | Moderate | Low | Fodder grass stabilizes sand dunes. |
| *Stipellula capensis* (Thunb.) Röser & Hamasha | Poaceae | 0 | 1 | 1 | 1 | Annual grass | Therophyte | High | Moderate | Low | Characteristic of Persain desert ecoregion |
| *Teucrium oliverianum* Ging. ex Benth. | Lamiaceae | 0 | 1 | 0 | 1 | Sub-Shrub | Chamaephyte | High | Low | Moderate | Ornamental, essential oil, medicinal |
| *Teucrium polium* L. | Lamiaceae | 0 | 1 | 0 | 1 | Sub-Shrub | Chamaephyte | High | Low | Low | Traditional medicinal. |
| *Tragus racemosus* (L.) All. | Poaceae | 0 | 1 | 0 | 0 | Annual grass | Therophyte | High | Moderate | Low | Aggressive weed. |
| *Tribulus terrestris* L. | Zygophyllaceae | 0 | 0 | 1 | 1 | Annual herb | Therophyte | Very High | Moderate | Low | Invasive noxious weed. Medicinal |
| *Trigonella anguina* Delile | Fabaceae | 0 | 1 | 1 | 0 | Annual herb | Therophyte | High | Moderate | Low | Ruderal invader competing with coastal species, Forage. Medicinal |
| *Trigonella spruneriana* Boiss. | Fabaceae | 0 | 1 | 1 | 0 | Annual herb | Therophyte | Moderate | Low | Low | Minor weed. Forage. Medicinal |
| *Trigonella stellata* Forssk. | Fabaceae | 0 | 1 | 0 | 0 | Annual herb | Therophyte | High | Low | Moderate | Good forage. Allelopathic |
| *Triticum aestivum* L. | Poaceae | 0 | 1 | 0 | 0 | Annual grass | Therophyte | Low | Low | Low | Staple crop |
| *Melilotus indicus* (L.) All. | Fabaceae | 0 | 1 | 0 | 1 | Annual herb | Therophyte | High | Moderate | Low | Nectar source for bees, Traditional Medicine, potential forage |
| *Tripleurospermum auriculatum* (Boiss.) Rech.f. | Asteraceae | 0 | 1 | 0 | 0 | Annual herb | Therophyte | Moderate |  | Low | Traditional Medicine |
| *Vachellia farnesiana* (L.) Wight & Arn. | Fabaceae | 0 | 0 | 1 | 1 | Shrub | Phanerophyte | High | Moderate | Moderate | Forage/Fodder. Wildlife nesting. Invasive |
| *Vachellia flava* (Forssk.) Kyal. & Boatwr. | Fabaceae | 0 | 0 | 1 | 1 | Tree | Phanerophyte | Very High | Moderate | Moderate | Fodder, Bee Nectar. Gum. Charcoal. Fiber |
| *Vachellia gerrardi* (Benth.) P.J.H.Hurter | Fabaceae | 1 | 1 | 1 | 1 | Tree | Phanerophyte | Very High | Moderate | Moderate | Fodder. Bee nectar. Firewood. Fencing. Nitrogen fixer |
| *Vachellia tortilis* (Forssk.) Galasso & Banfi | Fabaceae | 0 | 0 | 1 | 0 | Tree | Phanerophyte | Very High | High | Moderate | N-Fixer. Fodder. Arabic gum. |
| *Xanthium spinosum* L. | Asteraceae | 0 | 0 | 0 | 1 | Annual herb | Therophyte | Moderate | Moderate | Low | Noxious invasive weed |
| *Zilla spinosa* (L.) Prantl | Brassicaceae | 1 | 1 | 1 | 1 | Sub-Shrub | Chamaephyte | Very High | Moderate | Low | Traditional medicine. Pollinator |
| *Ziziphus nummularia* (Burm.f.) Whigt & Arnott | Rhamnaceae | 1 | 1 | 1 | 1 | Shrub | Phanerophyte | Very High | Moderate | Low | Medicinal. Nectar source. |
| *Zoegea purpurea* Fresen. | Asteraceae | 0 | 1 |  | 0 | Annual herb | Therophyte | High |  | Low | NA |
| *Zygophyllum bruguieri* (DC.) Christenh. & Byng | Zygophyllaceae | 0 | 0 | 1 | 1 | Sub-Shrub | Hemicryptophyte | Very High | High | Low | Medicinal |
| *Zygophyllum glutinosum* (Delile) Christenh. & Byng | Zygophyllaceae | 1 | 1 | 1 | 0 | Sub-Shrub | Chamaephyte | Very High | High | Low | NA |
| Total Species Recorded |  | 27 | 154 | 115 | 89 |  |  |  |  |  |  |

**Table S3** The Pearson Correlation Coefficient (r) of the soil variables and the dominant and important plants of the identified plant communities within Rawdhat Khuraym, Saudi Arabia, during the winter-spring season.

|  | **pH** | **EC** | **Na** | **K** | **Ca** | **Mg** | **SO4** | **HCO3-** | **Cl-** | **CaCO3** | **NO3-** | **NH4+** | **Available P** | **Clay** | **Silt** | **Sand** | **Field capacity** | **Bulk density** | **Porosity** |
| --- | --- | --- | --- | --- | --- | --- | --- | --- | --- | --- | --- | --- | --- | --- | --- | --- | --- | --- | --- |
| *A.fra* | 0.11 | -0.23 | -0.44 | -0.29 | -0.17 | -0.21 | -0.21 | -0.15 | 0.07 | -0.48 | -0.24 | -0.27 | -0.43 | -0.28 | -0.35 | 0.41 | -0.41 | 0.43 | -0.45 |
| *C.arv* | 0.05 | -0.25 | -0.28 | -0.11 | -0.23 | -0.30 | -0.07 | -0.31 | -0.50 | 0.55 | 0.08 | -0.04 | -0.20 | -0.14 | 0.58 | -0.41 | 0.25 | -0.31 | 0.24 |
| *C.pro* | -0.07 | 0.37 | 0.38 | 0.62 | 0.30 | 0.27 | 0.26 | 0.22 | -0.08 | 0.61 | 0.52 | 0.88 | 0.63 | 0.13 | 0.66 | -0.61 | 0.53 | -0.52 | 0.48 |
| *C.dec* | -0.79 | 0.90 | 0.75 | 0.72 | 0.92 | 0.83 | 0.95 | 0.77 | 0.41 | 0.02 | 0.64 | 0.21 | 0.63 | 0.63 | 0.03 | -0.30 | 0.47 | -0.45 | 0.50 |
| *C.spi* | 0.42 | -0.07 | 0.33 | 0.05 | -0.13 | -0.26 | -0.19 | -0.33 | 0.01 | 0.38 | -0.20 | 0.54 | 0.39 | 0.10 | 0.32 | -0.32 | 0.29 | -0.20 | 0.33 |
| *Car.oxy* | -0.55 | 0.74 | 0.72 | 0.51 | 0.78 | 0.59 | 0.77 | 0.82 | -0.06 | -0.04 | 0.77 | 0.13 | 0.58 | 0.53 | 0.14 | -0.34 | 0.47 | -0.41 | 0.39 |
| *Con.oxy* | -0.21 | -0.04 | 0.19 | -0.20 | -0.05 | 0.12 | 0.03 | 0.19 | -0.27 | -0.43 | -0.07 | -0.49 | -0.13 | 0.38 | -0.25 | 0.05 | 0.10 | -0.05 | -0.01 |
| *C.dac* | -0.30 | 0.44 | 0.34 | 0.66 | 0.37 | 0.28 | 0.22 | 0.14 | 0.59 | 0.43 | 0.00 | 0.79 | 0.65 | 0.37 | 0.26 | -0.38 | 0.40 | -0.44 | 0.50 |
| *H.ram* | 0.35 | -0.42 | -0.71 | -0.47 | -0.35 | -0.12 | -0.38 | -0.17 | 0.01 | -0.68 | -0.25 | -0.46 | -0.63 | -0.67 | -0.70 | 0.88 | -0.90 | 0.89 | -0.87 |
| *L.sha* | 0.05 | -0.22 | -0.19 | -0.46 | -0.17 | -0.29 | -0.32 | 0.18 | -0.27 | -0.61 | -0.24 | -0.62 | -0.26 | 0.06 | -0.50 | 0.40 | -0.31 | 0.33 | -0.38 |
| *M.par* | 0.62 | -0.37 | 0.03 | -0.24 | -0.43 | -0.48 | -0.39 | -0.57 | -0.10 | 0.53 | -0.21 | 0.23 | -0.06 | -0.31 | 0.40 | -0.20 | 0.06 | -0.05 | 0.08 |
| *P.min* | 0.09 | -0.09 | -0.42 | -0.17 | -0.03 | 0.03 | -0.15 | 0.19 | 0.01 | -0.47 | 0.02 | -0.27 | -0.23 | -0.39 | -0.53 | 0.61 | -0.61 | 0.58 | -0.58 |
| *P.ova* | 0.01 | -0.23 | -0.29 | -0.09 | -0.22 | -0.27 | -0.05 | -0.28 | -0.50 | 0.54 | 0.11 | -0.06 | -0.21 | -0.15 | 0.56 | -0.40 | 0.23 | -0.30 | 0.22 |
| *P.rep* | -0.71 | 0.86 | 0.73 | 0.66 | 0.89 | 0.76 | 0.91 | 0.83 | 0.22 | 0.01 | 0.76 | 0.19 | 0.61 | 0.56 | 0.09 | -0.31 | 0.46 | -0.43 | 0.44 |
| *P.und* | 0.21 | -0.31 | -0.41 | -0.37 | -0.25 | -0.22 | -0.24 | -0.25 | 0.05 | -0.49 | -0.27 | -0.32 | -0.51 | -0.34 | -0.36 | 0.45 | -0.45 | 0.48 | -0.49 |
| *R.str* | 0.59 | -0.61 | -0.73 | -0.62 | -0.57 | -0.32 | -0.53 | -0.43 | -0.08 | -0.41 | -0.31 | -0.48 | -0.74 | -0.85 | -0.51 | 0.79 | -0.89 | 0.86 | -0.85 |
| *R.spi* | 0.50 | -0.36 | -0.10 | -0.36 | -0.40 | -0.35 | -0.36 | -0.42 | 0.06 | 0.15 | -0.23 | -0.15 | -0.30 | -0.41 | 0.00 | 0.18 | -0.26 | 0.24 | -0.25 |
| *S.aeg* | 0.15 | -0.26 | -0.40 | -0.31 | -0.20 | -0.15 | -0.17 | -0.22 | 0.06 | -0.48 | -0.23 | -0.28 | -0.47 | -0.31 | -0.35 | 0.42 | -0.42 | 0.45 | -0.45 |
| *V.ger* | -0.39 | 0.15 | 0.19 | -0.06 | 0.16 | 0.21 | 0.12 | 0.46 | -0.13 | -0.49 | 0.01 | -0.48 | 0.02 | 0.45 | -0.38 | 0.13 | 0.04 | -0.03 | -0.03 |
| *Z.spi* | 0.17 | -0.28 | -0.45 | -0.34 | -0.22 | -0.16 | -0.20 | -0.22 | 0.06 | -0.52 | -0.24 | -0.31 | -0.51 | -0.34 | -0.39 | 0.47 | -0.47 | 0.50 | -0.50 |
| *Z.num* | -0.83 | 0.87 | 0.75 | 0.64 | 0.90 | 0.82 | 0.92 | 0.86 | 0.30 | -0.11 | 0.62 | 0.06 | 0.59 | 0.71 | -0.05 | -0.26 | 0.46 | -0.44 | 0.45 |

**Table S4** The Pearson Correlation Coefficient (r) of the soil variables and the dominant and important plants of the identified plant communities within Rawdhat Khuraym, Saudi Arabia, during the summer-fall season

|  | **pH** | **EC** | **Na** | **K** | **Ca** | **Mg** | **SO4** | **HCO3-** | **Cl-** | **CaCO3** | **NO3-** | **NH4+** | **Available P** | **Clay** | **Silt** | **Sand** | **Field capacity** | **Bulk density** | **Porosity** |
| --- | --- | --- | --- | --- | --- | --- | --- | --- | --- | --- | --- | --- | --- | --- | --- | --- | --- | --- | --- |
| *A.fat* | -0.09 | 0.28 | 0.23 | 0.61 | 0.17 | 0.30 | 0.13 | 0.16 | 0.04 | 0.57 | 0.57 | 0.86 | 0.48 | 0.05 | 0.67 | -0.53 | 0.45 | -0.46 | 0.38 |
| *C.pro* | -0.18 | 0.32 | 0.17 | 0.62 | 0.22 | 0.35 | 0.14 | 0.30 | 0.04 | 0.50 | 0.64 | 0.78 | 0.46 | 0.02 | 0.56 | -0.44 | 0.37 | -0.41 | 0.32 |
| *C.dec* | -0.86 | 0.87 | 0.67 | 0.66 | 0.91 | 0.74 | 0.95 | 0.72 | 0.93 | 0.07 | 0.62 | 0.13 | 0.53 | 0.73 | 0.02 | -0.30 | 0.45 | -0.46 | 0.38 |
| *C.spi* | -0.08 | 0.22 | 0.25 | 0.51 | 0.09 | 0.30 | 0.11 | 0.15 | -0.10 | 0.78 | 0.58 | 0.69 | 0.37 | 0.01 | 0.81 | -0.63 | 0.50 | -0.53 | 0.46 |
| *C.oxy* | 0.62 | -0.28 | 0.26 | -0.12 | -0.40 | -0.39 | -0.32 | -0.56 | -0.52 | 0.71 | -0.26 | 0.34 | 0.20 | 0.01 | 0.68 | -0.54 | 0.42 | -0.37 | 0.47 |
| *C.pil* | -0.51 | 0.20 | -0.08 | -0.04 | 0.30 | 0.40 | 0.35 | 0.36 | 0.48 | -0.51 | 0.00 | -0.44 | -0.28 | 0.14 | -0.45 | 0.29 | -0.20 | 0.21 | -0.29 |
| *C.dac* | 0.39 | 0.05 | 0.58 | 0.07 | -0.03 | -0.14 | -0.06 | -0.23 | -0.19 | 0.51 | -0.32 | 0.43 | 0.55 | 0.50 | 0.48 | -0.56 | 0.58 | -0.53 | 0.66 |
| *E.cil* | -0.22 | 0.10 | -0.04 | -0.04 | 0.12 | 0.31 | -0.03 | 0.53 | -0.11 | -0.13 | -0.08 | -0.22 | 0.02 | 0.20 | -0.23 | 0.10 | -0.06 | -0.04 | 0.05 |
| *L.sha* | -0.03 | -0.23 | -0.49 | -0.33 | -0.18 | -0.02 | -0.31 | 0.26 | -0.28 | -0.45 | -0.22 | -0.49 | -0.38 | -0.25 | -0.53 | 0.51 | -0.50 | 0.41 | -0.40 |
| *P.min* | -0.09 | 0.28 | 0.23 | 0.61 | 0.17 | 0.30 | 0.13 | 0.16 | 0.04 | 0.57 | 0.57 | 0.86 | 0.48 | 0.05 | 0.67 | -0.53 | 0.45 | -0.46 | 0.38 |
| *P.und* | 0.21 | -0.48 | -0.47 | -0.52 | -0.45 | -0.24 | -0.31 | -0.37 | -0.26 | -0.25 | -0.37 | -0.50 | -0.69 | -0.45 | -0.17 | 0.31 | -0.38 | 0.42 | -0.44 |
| *R.str* | 0.24 | -0.47 | -0.73 | -0.47 | -0.40 | -0.57 | -0.41 | -0.36 | -0.21 | -0.63 | -0.14 | -0.53 | -0.61 | -0.73 | -0.66 | 0.80 | -0.81 | 0.79 | -0.79 |
| *S.cap* | 0.14 | -0.47 | -0.66 | -0.52 | -0.38 | -0.34 | -0.30 | -0.38 | -0.05 | -0.71 | -0.36 | -0.56 | -0.76 | -0.57 | -0.58 | 0.68 | -0.68 | 0.73 | -0.77 |
| *V.ger* | -0.24 | 0.10 | -0.06 | -0.05 | 0.13 | 0.32 | -0.01 | 0.54 | -0.08 | -0.17 | -0.08 | -0.26 | -0.01 | 0.19 | -0.26 | 0.13 | -0.09 | -0.01 | 0.02 |
| *Z.spi* | 0.05 | -0.33 | -0.47 | -0.40 | -0.25 | -0.13 | -0.19 | -0.21 | 0.01 | -0.58 | -0.35 | -0.43 | -0.59 | -0.34 | -0.44 | 0.47 | -0.47 | 0.51 | -0.56 |
| *Z.num* | -0.80 | 0.74 | 0.51 | 0.49 | 0.79 | 0.79 | 0.72 | 0.90 | 0.65 | -0.01 | 0.42 | -0.03 | 0.43 | 0.71 | -0.11 | -0.19 | 0.33 | -0.40 | 0.35 |

**
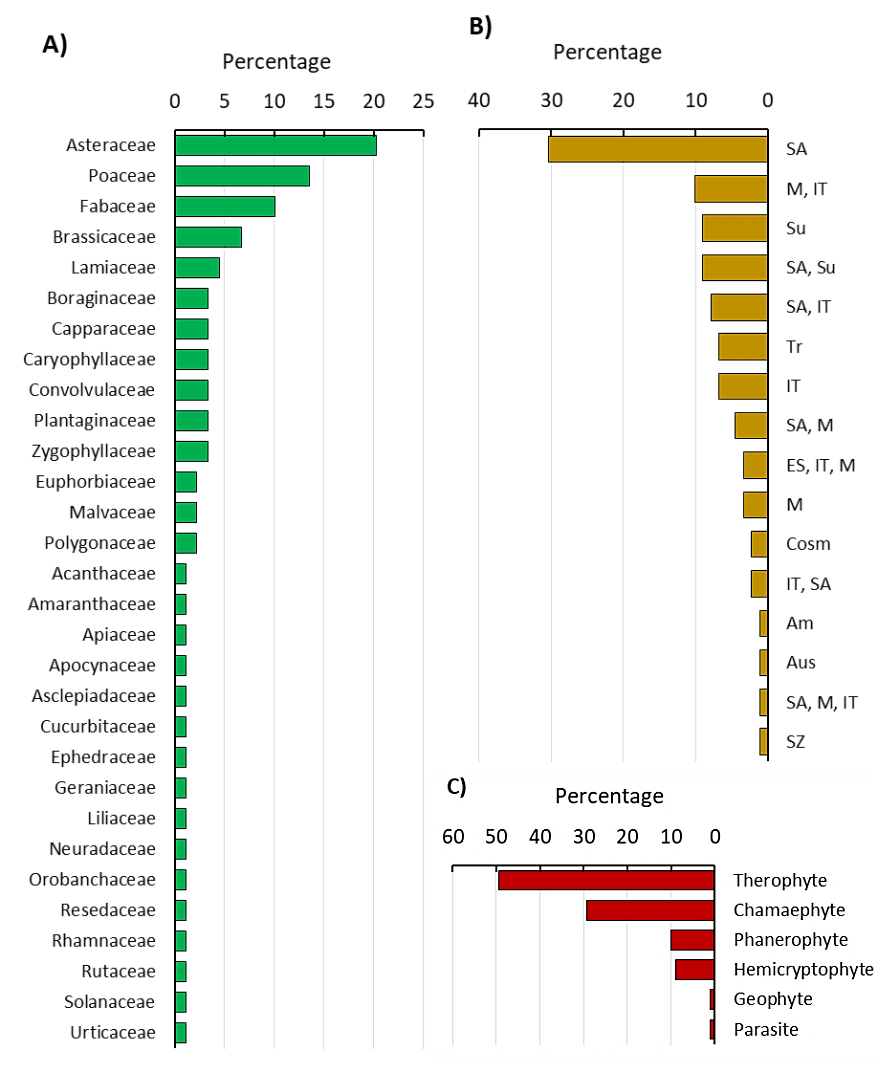
**

**Fig. S1** Floristic composition of the studied sites within Rawdhat Khuraym, Saudi Arabia. **A**) represented families, **B**) chorotype spectra, and **C**) life forms.


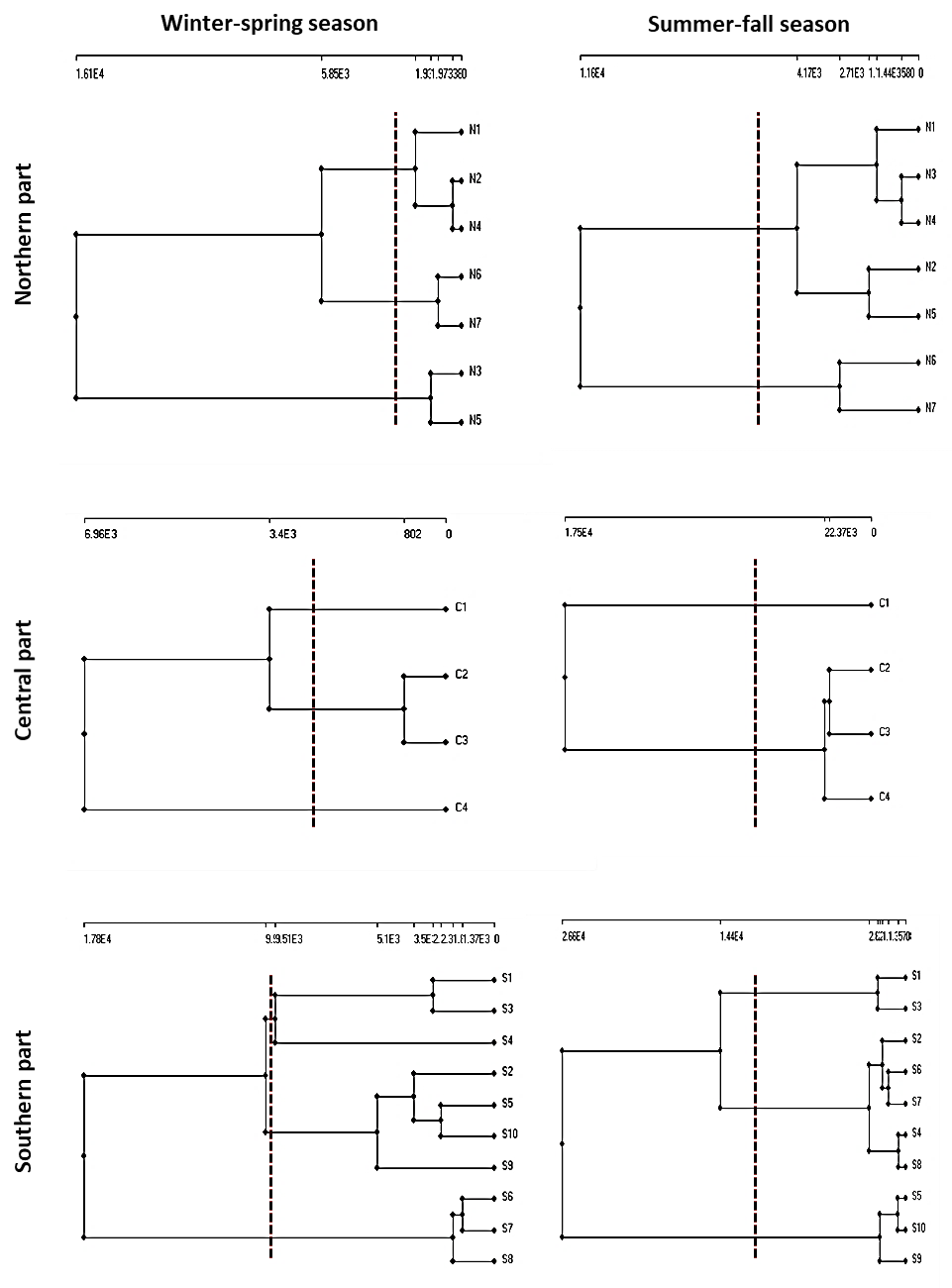


**Fig. S2** Hierarchical cluster analysis of the studied sites of Rawdhat Khuraym, Saudi Arabia, during the winter-spring and summer-fall seasons.


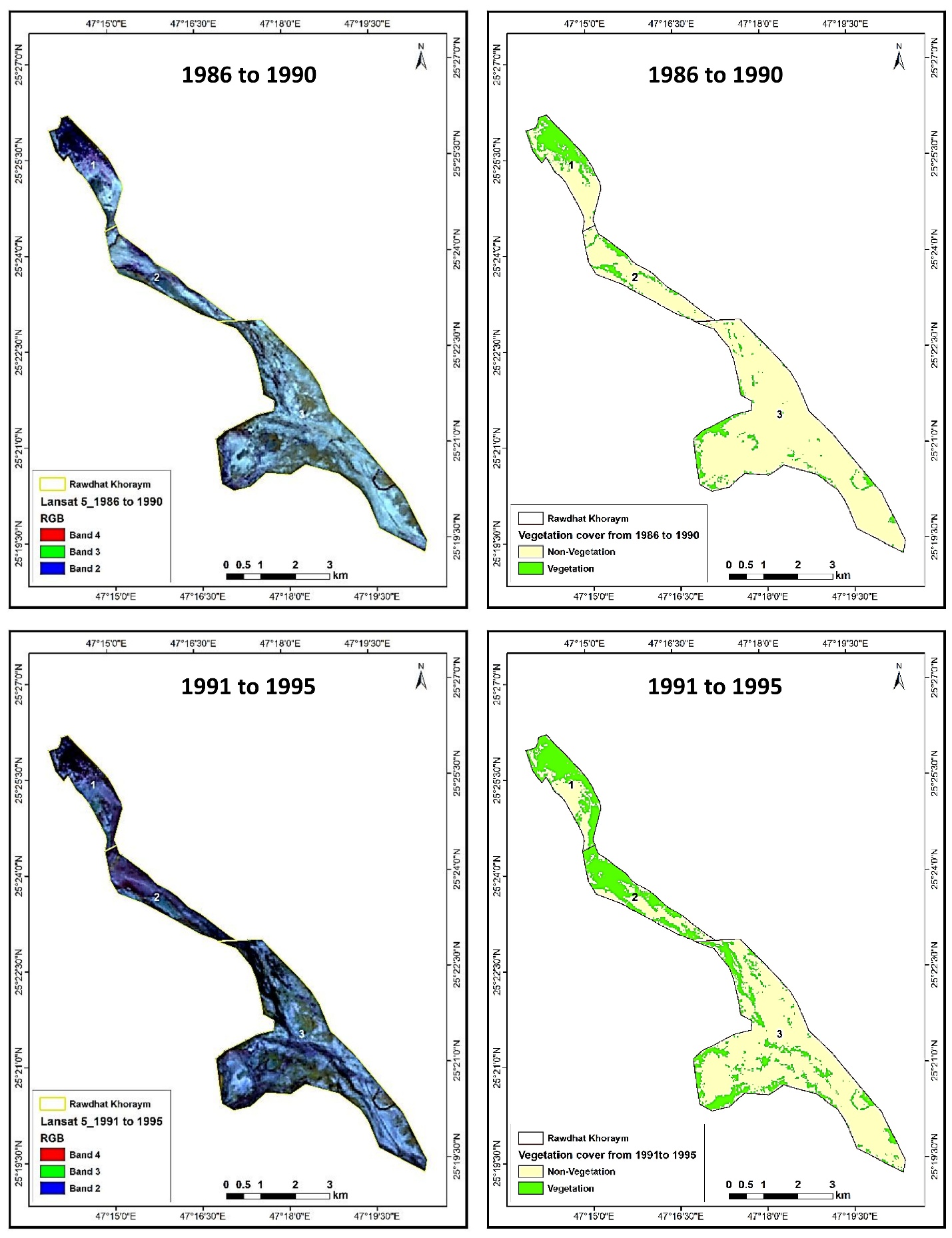
**Fig. S3** Sentinel 2 false color composite image and extracted vegetation cover during the period from 1986 to 1995.


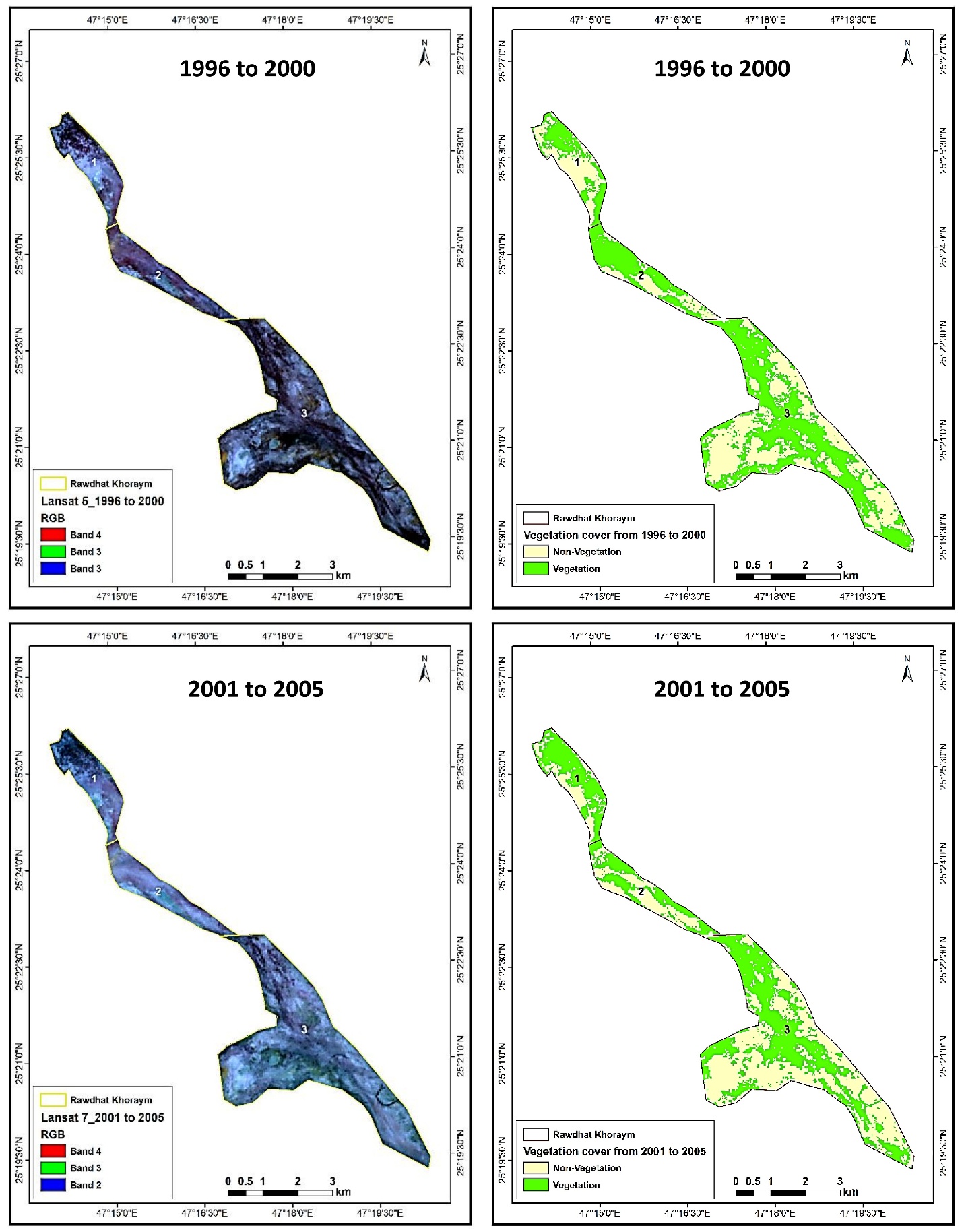


**Fig. S4** Sentinel 2 false color composite image and extracted vegetation cover during the period from 1996 to 2005.


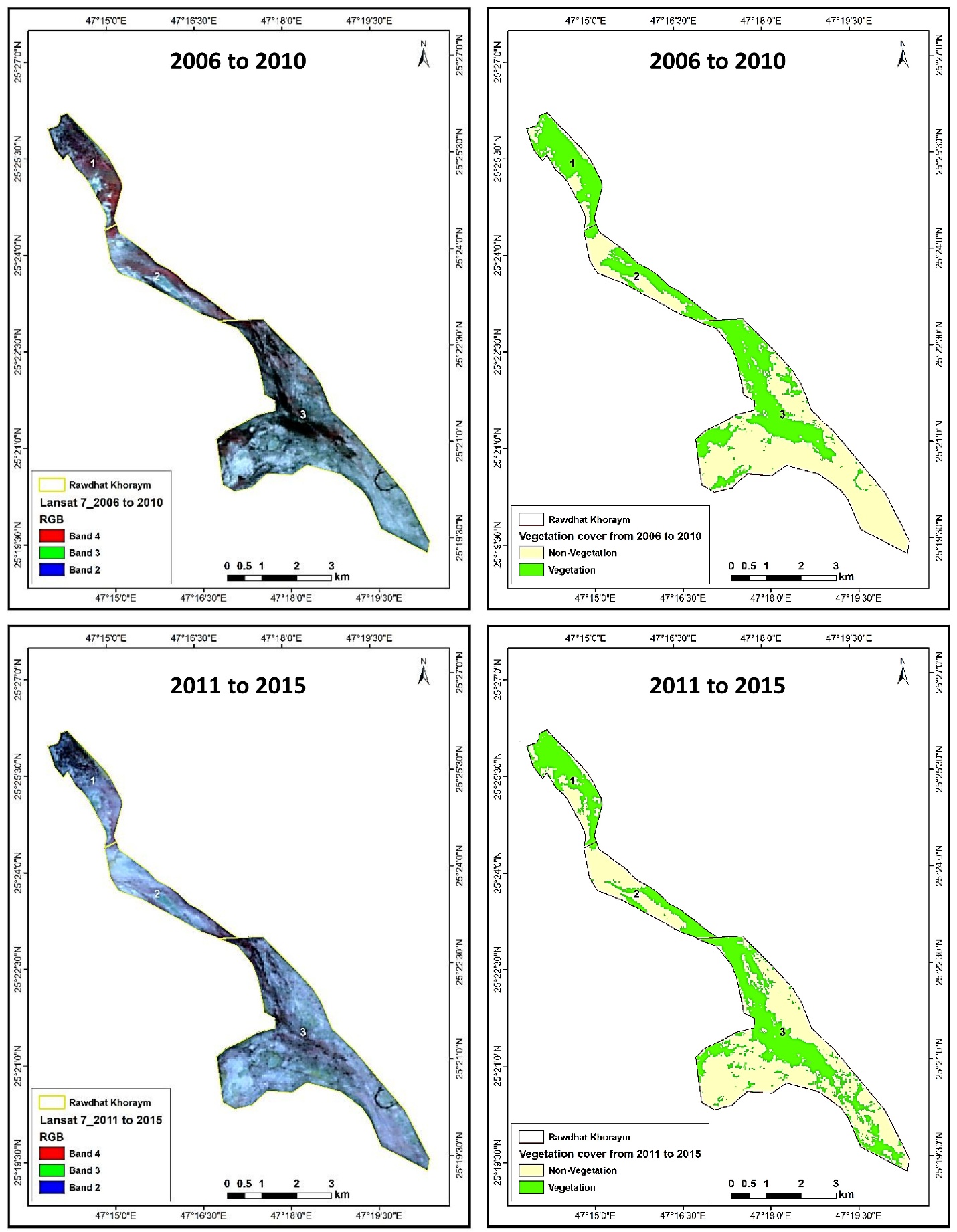


**Fig. S5** Sentinel 2 false color composite image and extracted vegetation cover during the period from 2006 to 2015.


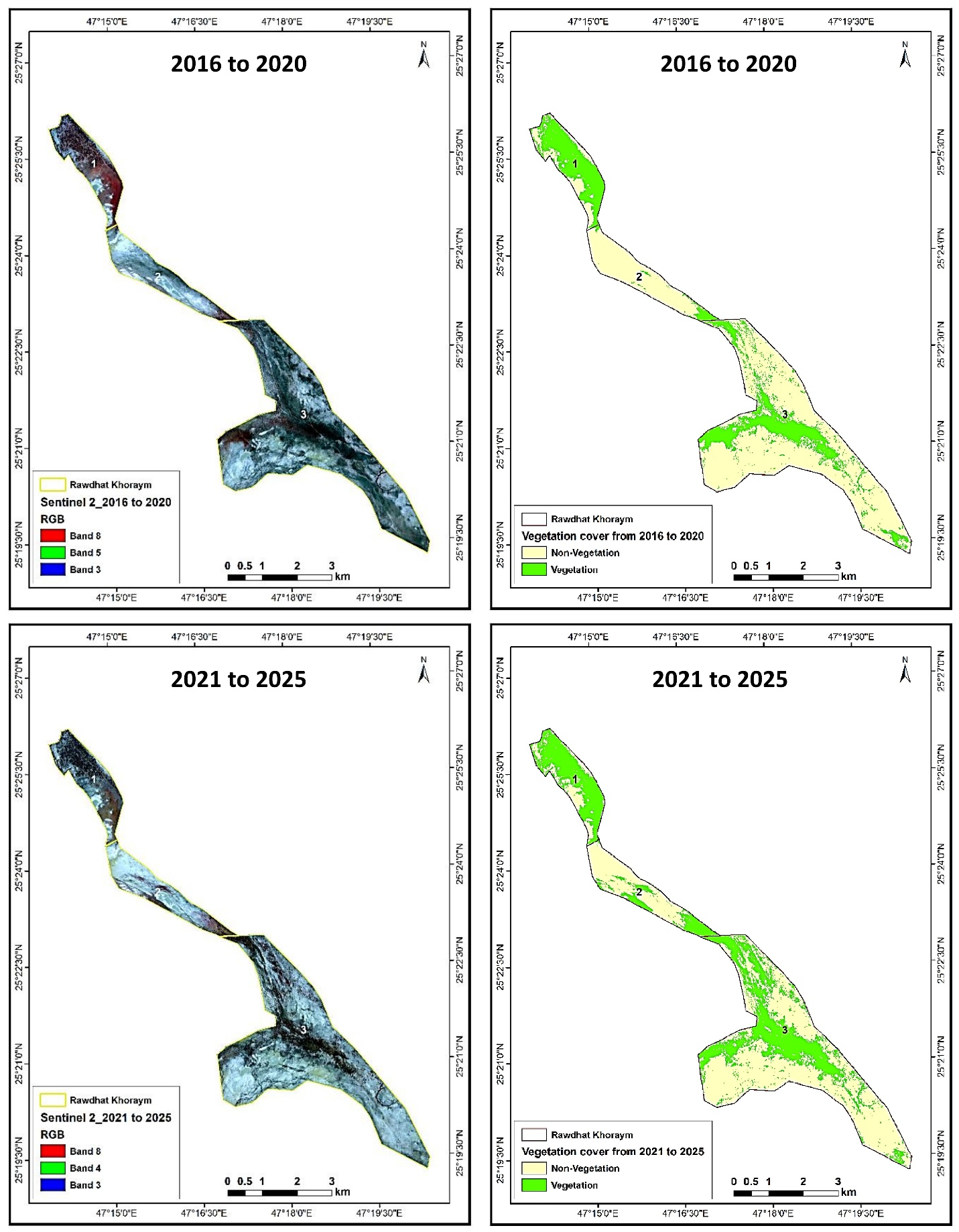


**Fig. S6** Sentinel 2 false color composite image and extracted vegetation cover during the period from 2016 to 2025.


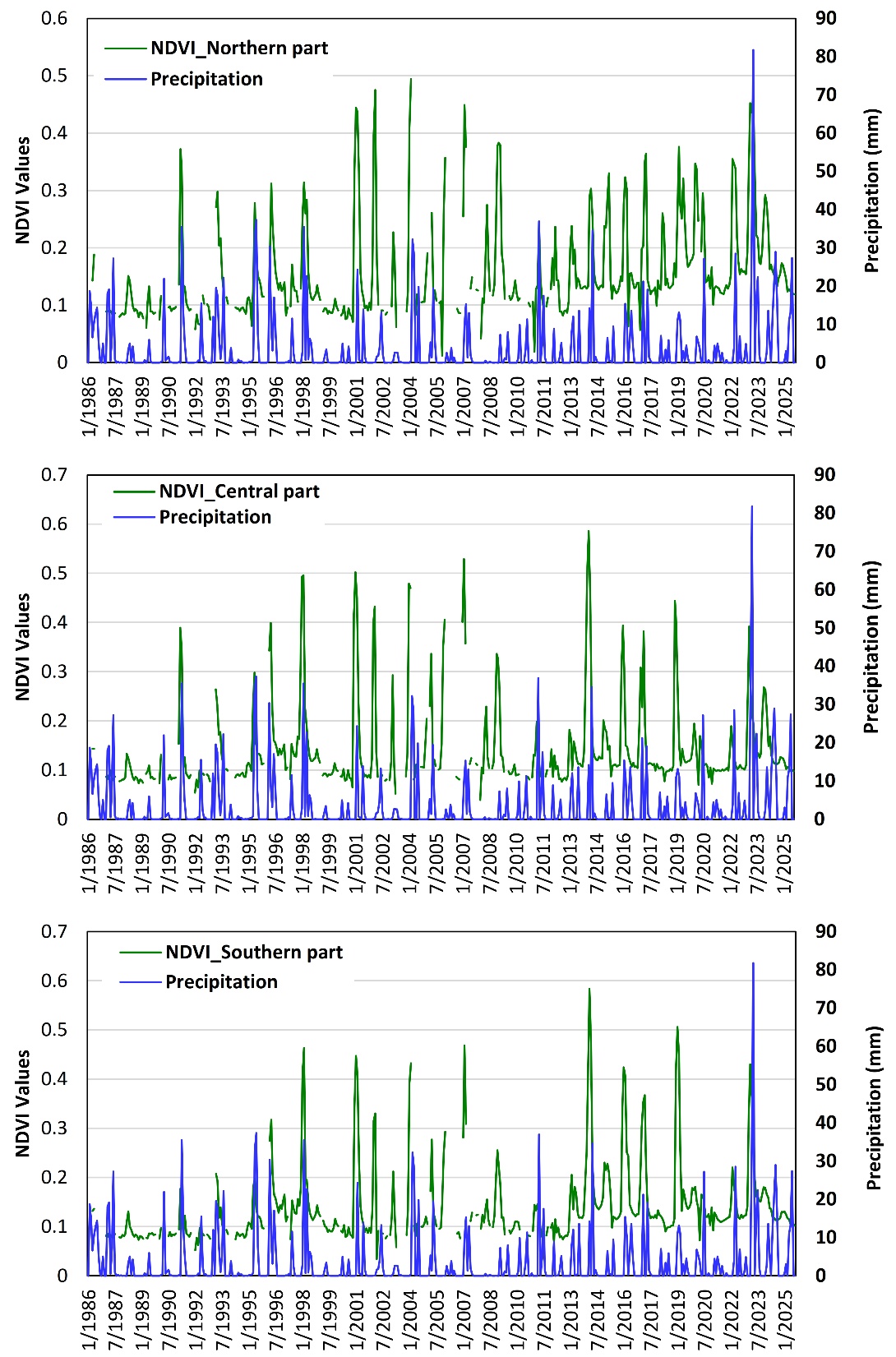


**Fig. S7** NDVI values and monthly precipitation in the different studied parts of Rawdhat Khuraym from 1986 to 2025 at two months' time lag.
